# The antioxidant response favors *Leishmania* parasites survival, limits inflammation and reprograms the host cell metabolism

**DOI:** 10.1371/journal.ppat.1009422

**Published:** 2021-03-25

**Authors:** Marta Reverte, Remzi Onur Eren, Baijayanti Jha, Chantal Desponds, Tiia Snäkä, Florence Prevel, Nathalie Isorce, Lon-Fye Lye, Katherine L. Owens, Ulisses Gazos Lopes, Stephen M. Beverley, Nicolas Fasel

**Affiliations:** 1 Department of Biochemistry, University of Lausanne, Epalinges, Switzerland; 2 Department of Molecular Microbiology, School of Medicine, Washington University, St. Louis, Missouri, United States of America; 3 Carlos Chagas Filho Biophysics Institute, Center of Health Science, Federal University of Rio de Janeiro, Rio de Janeiro, Brazil; INRS - Institut Armand Frappier, CANADA

## Abstract

The oxidative burst generated by the host immune system can restrict intracellular parasite entry and growth. While this burst leads to the induction of antioxidative enzymes, the molecular mechanisms and the consequences of this counter-response on the life of intracellular human parasites are largely unknown. The transcription factor NF-E2-related factor (NRF2) could be a key mediator of antioxidant signaling during infection due to the entry of parasites. Here, we showed that NRF2 was strongly upregulated in infection with the human *Leishmania* protozoan parasites, its activation was dependent on a NADPH oxidase 2 (NOX2) and SRC family of protein tyrosine kinases (SFKs) signaling pathway and it reprogrammed host cell metabolism. In inflammatory leishmaniasis caused by a viral endosymbiont inducing TNF-α in chronic leishmaniasis, NRF2 activation promoted parasite persistence but limited TNF-α production and tissue destruction. These data provided evidence of the dual role of NRF2 in protecting both the invading pathogen from reactive oxygen species and the host from an excess of the TNF-α destructive pro-inflammatory cytokine.

## Author summary

When entering host cells, *Leishmania* parasites have evolved ingenious ways to resist an array of microbicidal mechanisms, such as oxidative burst, generated by the host immune system to restrict parasite growth. Recent studies have highlighted the NF-E2-related factor 2 (NRF2) as being responsible for conferring resistance to oxidative stress in *Leishmania* infection by the induction of cytoprotective genes. However, the mechanisms by which the antioxidant response is induced by parasitic infection and the impact on parasite survival are largely unknow. Here, we showed that NRF2 was strongly upregulated in *Leishmania* infection and participated in *Leishmania* parasite survival. We described that NRF2 expression was dependent on NADPH oxidase 2 and SRC family of protein tyrosine kinases (SFKs) signaling, leading to it being translocated into the nucleus and activating specific downstream genes. Furthermore, we observed that cell surface contact by *Leishmania* and phagocytosis were important to reprogram host cell metabolism in an NRF2-dependent manner. In addition, when we focused on *Leishmania guyanensis* parasites, which bear an endosymbiotic dsRNA virus known to exacerbate the disease and dissemination of the infection, we concluded that NRF2 limited inflammation and pathology by controlling the levels of the anti-*Leishmania* cytokine TNF-α which can cause tissue destruction in patients suffering of mucocutaneous leishmaniasis. Thus, the activation of the host oxidative stress pathway is likely an important subversion mechanism that *Leishmania* parasites use to promote their own persistence but also a protective mechanism for the host to limit inflammation in co-infections or in immunocompromised situations.

## Introduction

Macrophages are myeloid immune cells specialized in the recognition and elimination of invading pathogens [1,2]. They can, however, be colonized by protozoan parasites such as *Toxoplasma gondi*, *Trypanosoma cruzi* or *Leishmania* (*L*.) *spp*., offering them an environment to complete their life cycle and multiply. Among these different human pathogens, *L*. *spp*. are a genus of obligate protozoan parasites causing a broad spectrum of neglected tropical diseases known as leishmaniasis [3].

Cutaneous leishmaniasis (CL) is the most frequent infection and generally manifests as a self-healing lesion localized at the inoculation site of parasites by female sand flies and is caused by different *L*. *spp*. In South America, up to 10% of the cases progress to persistent chronic inflammation resulting in distant secondary lesions leading to mucocutaneous leishmaniasis (MCL), with destruction of the mucosal tissues [4,5]. Such aggressive symptomatic outcomes may be due to co-infections with other viruses [6,7] or the presence of a cytoplasmic double stranded RNA (dsRNA) virus, *Leishmania* RNA virus 1 (LRV1) nested mainly within *L*. *braziliensis (Lbr*LRV1+*)* or *L*. *guyanensis (Lgy) (Lgy*LRV1+*)* parasites. As shown in murine models, the dsRNA genome of LRV1 is recognized by the host endosomal Toll-like receptor-3 (TLR-3) [8,9], inducing tumor necrosis factor-α (TNF-α) expression and a potent type I interferon (IFN-I) anti-viral immune response leading to IL-6 driven hyperinflammation [6], disease exacerbation and IL-17-dependent metastasis in IFN-γ deficient (*Ifng*^-/-^) mice, which can be correlated with MCL *Lgy* infected patients showing high IL-17 level but low IFN-γ levels [10]. MCL patients have increased levels of TNF-α produced by specific intermediate monocytes [11] that could explain destruction of mucosal tissues observed in these patients [12].

At the inoculation site, *L*. parasites enter human skin macrophages as flagellated promastigotes via receptor-mediated phagocytosis. Several cell surface receptors facilitate parasite entry such as complement receptors (CR1 and CR3), mannose receptor (MR), Fc gamma receptors (FcγR) and Toll-like receptors (TLRs) [13]. Entry into macrophages is a very rapid process since, after 10 minutes of incubation, Rab7 is already detected on *Leishmania*-containing phagosomes [14]. Once in macrophages, *L*. parasites reside in phagolysosomal compartments, which are characterized by an increase in temperature and decrease in acidic conditions triggering the differentiation of promastigotes into amastigotes, which present a shorter and non-motile flagellum [15,16]. Of note, promastigotes and amastigotes differ in the type of receptors and the signaling pathway they use to enter the cell [13]. Efficient phagocytosis requires Abl family kinases which can be activated by SRC Family Kinases (SFK) [17,18].

Phagocytosis triggers assembly of the NADPH oxidase 2 (NOX2) complex in macrophages resulting in reactive oxygen species (ROS) production. Disabling NOX2 assembly is a strategy developed by various pathogens to subvert exposure to oxidants. In *Leishmania* infections, inhibition of phagolysosome maturation is characterized by an impaired assembly of NOX2 [19–22]. In addition, the anti-*Leishmania* response in macrophages also consists of the production of reactive nitrogen species (RNS) by the inducible nitric oxide synthase (iNOS). Evidently, *Leishmania* parasites could benefit from the decrease of the oxidative burst due to the induction of the host antioxidant response, ultimately favoring their survival.

NF-E2-related factor 2 (NRF2) is a master regulator of antioxidant response genes [23]. *Nrf2* deficient (*Nrf2*^-/-^) mice are susceptible to a broad range of xenobiotics and diseases with oxidative pathology [24]. Under basal conditions, NRF2 is bound to the homodimeric Kelch-like ECH-associated protein 1 (KEAP1) [23]. KEAP1 sequesters NRF2 in the cytosol and acts as a substrate adaptor for the ubiquitination on NRF2 and its degradation by the proteasome. Upon exposure to electrophilic chemicals, such as *tert*-butyl hydroquinone (tBHQ) [25], or ROS, KEAP1 is inactivated by electrophilic and/or oxidative modifications and NRF2 is stabilized and translocated into the nucleus. Phosphorylation of NRF2 occurs at different sites and regulates its level in the nucleus [26–29] where it forms a heterodimer with the small musculoaponeurotic fibrosarcoma (sMAF) transcription factor and binds to the defined antioxidant-responsive element (ARE), present in its own promoter activating its own transcription, and in the promoters of several NRF2 target genes, such as NADPH quinone oxidoreductase 1 (*Nqo1*) and Heme oxygenase 1 (*Hmox1*), coding for NQO1 and HO-1 proteins, respectively [30,31]. Expression of NRF2-downstream genes can be increased by co-activation by activating transcription factor 4 (ATF4), which is induced in stress situations for example by protein kinase R-like ER kinase (PERK) and eukaryotic translation initiation factor 2 (eIF2α) [32].

Although the main control of NRF2 stability is exerted by KEAP1, other activator proteins could be implicated in NRF2 activation including iNOS, important in protecting the host against *Leishmania* [33,34], pathways implicating TLR agonists, c-SRC and NADPH oxidase [35] or the protein kinase C δ (PKCδ), which is regulated by phosphorylation by a non-receptor tyrosine protein kinase SRC (SRC). PKCδ could in turn be implicated in NRF2 phosphorylation on serine 40 thus inducing NRF2 nuclear translocation and transcriptional activation of *Hmox1* after oxidant stimulus [29].

Although NRF2 is likely activated following infection with *L*. *donovani* [36,37], *L*. *chagasi* [38] or *L*. *braziliensis* [39], as evidenced by increased expression of NRF2-regulated genes such as *Hmox1*, the underlying signaling pathway leading to NRF2 activation in *L*. *spp*. infection is still not well defined, apart from *L*. *amazonensis* infection. In this latter case, the NRF2 signaling pathway depends on a dsRNA induced kinase PKR and PI3K/AKT pathway [40]. Furthermore, in *L*. *amazonensis* infection, NRF2 activation can also be induced by phosphorylation of PERK culminating in increased expression of NRF2-downstream genes such as *Hmox1*, which has antioxidant and anti-inflammatory properties [41]. Differences in NRF2 activation could exist between *L*. *spp*. For example, more ROS are produced in *L*. *major* infected cells than in *L*. *amazonensis* infection suggesting that *L*. *amazonensis* could more efficiently control oxidative stress possibly via NRF2 [42]. Furthermore, killing of intracellular *Leishmania* parasites can differ between species. For example, *L*. *guyanensis* is more susceptible than *L*. *amazonensis*, whereas other species inhibit ROS generation [43,44]. In this respect, a proteomic study describes a higher HO-1 level in *L*. *amazonensis* infected cells than in *L*. *major* infected cells [45]. Thus far, it is only in *L*. *amazonensi*s infection where NRF2 activation leads to parasite survival and disease progression [40].

Additionally, NRF2 activation limits inflammation by decreasing nuclear factor kappa B (NF-κB)-activated proinflammatory cytokines such as TNF-α, iNOS, COX-2 or IL-6 [46–50]. A loss of control of NRF2 onto the NF-κB pathway could be relevant in hyperinflammation situations which occur in inflammatory leishmaniases such as MCL displaying high TNF-α levels in secondary lesions [51]. Finally, NRF2 is also recognized as a crucial player in reprogramming cancer cell metabolism [50] but whether NRF2 plays a role in the metabolism of macrophages infected by *Leishmania* remains unknown.

In this study, we investigated the significance of the NRF2 pathway in *Leishmania* infection, by describing the molecular mechanism underlying the trigger of NRF2 signaling in macrophages by *Leishmania* and its subsequent protective role in exacerbated inflammatory cases of leishmaniasis driven by LRV1 present mainly in South America. This in turn eludes to why such a series of triggers could lead to IL-6 and TNF-α production [8], dissemination of the infection [10] and culminate in pathological situations as observed in MCL [11,12,52].

## Results

### The infection of macrophages with *Leishmania spp*. leads to upregulation of NRF2 pathway

**Fig 1:**
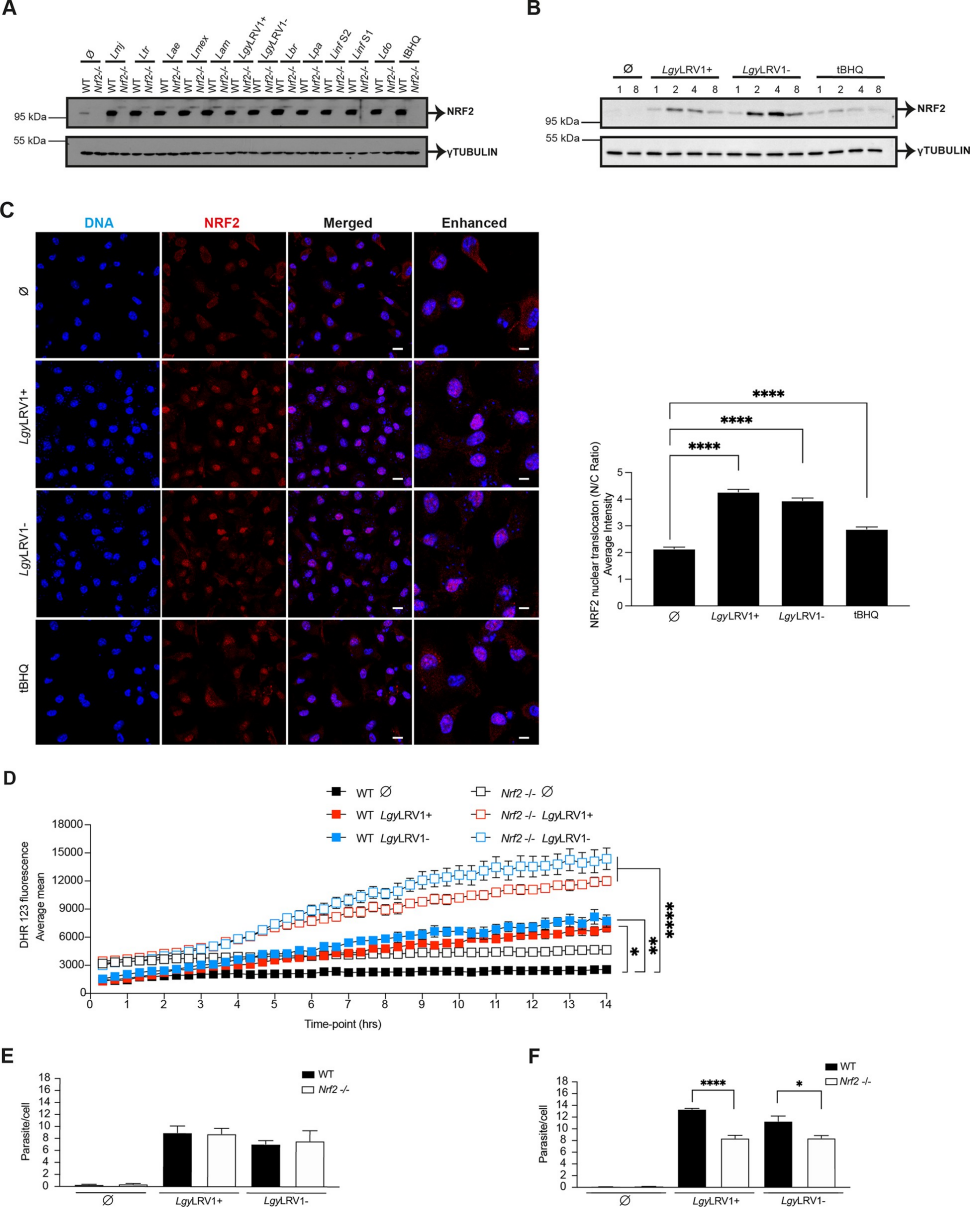
The infection of macrophages with *Leishmania spp*. leads to upregulation of NRF2 pathway. A) Western blot analysis of WT and *Nrf2*^-/-^ BMDMs infected with *Lmj*, *Ltr*, *Lae*, *Lmex*, *Lam*, *Lgy*LRV1+, *Lgy*LRV1-, *Lbr*, *Lpa*, *Linf* S1, *Linf* S2 or *Ldo* parasites for 4 hrs. Analysis of non-treated (Ø) or tBHQ-treated (10 μM) cells were performed concomitantly. Cells lysates were tested with anti-NRF2 and anti-γTUBULIN antibodies. B) WT BMDMs infected with *Lgy*LRV1+ or *Lgy*LRV1- parasites for 1, 2, 4 or 8 hrs, or stimulated with medium (Ø), or tBHQ (10 μM) were analyzed by immunoblotting with anti-NRF2 and anti-γTUBULIN antibodies. C) Immunofluorescence and quantification of NRF2 nuclear translocation expressed as nucleus/cytoplasm (N/C) ratio in WT BMDMs. Cells were stained with NRF2 (red), DAPI (blue) after 8 hrs of infection with *Lgy*LRV1+ or *Lgy*LRV1- parasites, or stimulated with medium (Ø), or tBHQ (10 μM) and imaged at 63x using a confocal microscope. Scale bar represents 10 and 40 μm for merged and enhanced images, respectively. NRF2 nuclear translocation was quantified for each cell (N = 100–160) using IMARIS software. D) Intracellular levels of ROS in CFSE labelled WT and *Nrf2*^-/-^ cells infected with *Lgy*LRV1+ or *Lgy*LRV1- parasites or treated with medium (Ø) assessed using DHR 123 time-lapse microscopy. Cells were imaged at 20x for 14 hrs. ROS quantification was determined by Image J software using CFSE and DHR 123 staining. E) and F) Intracellular parasite load in WT and Nrf2^-/-^ cells infected with *Lgy*LRV1+ or *Lgy*LRV1- parasites at 8 hrs (E) or 24 hrs (F) stained with DAPI and imaged at 40x using a high-content microscope. Parasite and cell quantification were assessed using MetaXpress software. Representative blots (A-B) from three independent experiments are shown. Representative images (C) and quantification (C and D) from one of three independent experiments are shown. Data are expressed as mean ± SEM. Statistical significance was calculated by performing two-way ANOVA analysis with Bonferroni’s post-test (D) and unpaired Student’s t test (C and F). * p < 0.05, ** p < 0.01 and **** p < 0.0001. See also S1 Fig.

To determine whether activation of the NRF2 pathway occured with different *L*. *spp*., bone marrow derived macrophages (BMDMs) from C57BL/6 wild-type (WT) mice were infected with different *L*. species (*spp*.) for 4 hours (hrs) including *Lgy* harboring, or not, the LRV1 endosymbiont. Cell lysates were tested for NRF2 expression by immunoblotting (Fig 1A). We observed that all tested *L*. *spp*. showed similar levels of NRF2 protein at 4 hrs and NRF2 activation was sustained at 8 hrs post-infection, although we could observe some differences in expression level mainly in species inducing visceral leishmaniasis (S1A Fig). As expected, no expression of NRF2 was detected in *Nrf2*^-/-^ macrophages and *tert*-Butylhydroquinone (tBHQ) served as an inducer of NRF2 accumulation (Figs 1A and S1A). Gene expression of the known NRF2-regulated gene *Nqo1* [53] in WT cells confirmed that the different *L*. *spp*. promoted activation of the NRF2 pathway although some intrinsic differences in the level of expression could be observed between species (S1B Fig). Differences in expression level of NRF2 or *Nqo1* between various *L*. *spp*. were not due to different levels of infection as we can conclude from S1C Fig. We then focused our study on *Lgy* parasites with LRV1 (*Lgy*LRV1+) or without (*Lgy*LRV1-). We examined the kinetics of NRF2 activation in WT BMDMs infected with *Lgy*LRV1+ and *Lgy*LRV1- parasites and monitored the expression of NRF2 protein 1, 2, 4 and 8 hrs post-infection. NRF2 protein peaked in both *Lgy*LRV1+ and *Lgy*LRV1- parasites at 2–4 hrs (Fig 1B). These findings were validated with macrophages from *Nrf2*^-/-^ mice (S1D Fig), which contain a β-galactosidase (LacZ) reporter gene replacing the b-Zip region of the *Nrf2* gene [23], which allows the measurement of NRF2 activation using anti β-galactosidase antibody. We observed that both *Lgy*LRV1+ and *Lgy*LRV1- parasites induced β-galactosidase expression at 2–4 hrs post-infection (S1D Fig). A major increase in expression of NRF2 at mRNA level as determined by qRT-PCR occurred at 4 hrs post-infection with both *Lgy*LRV1+ and *Lgy*LRV1- parasites or after tBHQ treatment (S1E Fig). Thus, the time course of expression of NRF2 post-infection was similar at the mRNA and protein level. These data pointed to an upregulation of the expression of NRF2 by increased transcription of the *Nrf2* gene leading to increased NRF2 protein levels in infected or treated cells since we observed a similar pattern of expression in WT cells or in *Nrf2*^-/-^ mice in which a β-galactosidase (LacZ) reporter gene replaced *Nrf2* [23]. Though we cannot formally exclude other regulatory mechanisms such as differences in mRNA stability.

To confirm the induction of the NRF2 pathway in *Lgy* parasites, we visualized and quantified nuclear translocation of the NRF2 protein in WT BMDMs using confocal microscopy and IMARIS software, respectively. Both *Lgy*LRV1+ and *Lgy*LRV1- significantly promoted the nuclear translocation of NRF2 expressed as nucleus/cytoplasm ratio (Fig 1C) independently of the presence of LRV1 at 8 hrs post-infection. As shown in S1F Fig, translocation of NRF2 into the nucleus and activation of the NRF2 pathway induced the expression of two known NRF2-target genes, *Hmox1* and *Nqo1* (S1F Fig). Upregulation of these two genes paralleled upregulation of NRF2 and was maximal at 4 hrs post-infection. Increased expression of the target genes was still maintained at some level 8 hrs post-infection. We confirmed the LRV1 and TLR-3 independent activation of NRF2 by infecting WT and TLR-3 deficient (*Tlr3*^-/-^) macrophages with *Lgy*LRV1+ and *Lgy*LRV1-, or treated them with tBHQ or poly I:C, an agonist of TLR-3. We observed no difference in NRF2 expression between WT and *Tlr3*^-/-^ cells infected with *Lgy*LRV1+ and *Lgy*LRV1- (S1G Fig). To investigate whether NRF2 activation by *Leishmania* was dependent on other TLRs, we infected BMDMs from mice deficient in myeloid differentiation primary response gene 88 (*MyD88*^-/-^), which is implicated as an adaptor molecule for signal transmission in all the TLRs except for TLR-3. *MyD88*^-/-^ BMDMs were infected with *Lgy*LRV1+, *Lgy*LRV1-, *Lmj* or *Linf* S1 and *Linf* S2 parasites, or stimulated with tBHQ. As shown in S1H Fig, NRF2 was similarly expressed in WT and in *MyD88*^-/-^ cells infected with different *L*. *spp*. Thus, NRF2 activation and expression did not depend on MyD88 (S1H Fig). We also compared expression of NRF2-target genes, *Hmox1* and *Nqo1*, in WT and *Tlr3* ^-/-^ cells at 8 hrs post-infection. As shown, we observed that their expression did not rely on LRV1-TLR-3 activation in *Lgy* infected macrophages (S1I Fig).

The function of NRF2 in regulating antioxidant defense has been widely reported [54]. Therefore, we evaluated its role in ROS detoxification in an *Lgy* infection model using WT and *Nrf2*^-/-^ BMDMs and measuring ROS accumulation with DHR 123 staining. Our results confirmed that NRF2 was involved in the induction of an antioxidant response in *Lgy* infection as *Nrf2*^-/-^ cells showed greater accumulation of ROS in both *Lgy*LRV1+ and *Lgy*LRV1- (Fig 1D). We could exclude that differences in ROS accumulation were due to differences in host cell survival since both WT and *Nrf2*^-/-^ infected, and non-infected cells, showed similar cell viability as determined by the live cell marker CFSE (S1J Fig). Since oxidative burst is one of the main microbicidal mechanisms against phagocytosed microorganisms [55], we investigated if ROS detoxification by the NRF2 pathway had an effect on parasite load in *Lgy* infection. At early time-points, parasite burden was similar between WT and *Nrf2*^-/-^ cells in *Lgy* infection (Fig 1E), showing a similar rate of infection but no effect of the lack of NRF2 expression on parasite survival in the presence of higher ROS levels. In contrast, *Nrf2*^-/-^ cells presented lower parasite burden in both *Lgy*LRV1+ and *Lgy*LRV1- infection at 24 hrs post-infection (Fig 1F). These results imply that the NRF2 pathway was activated in *Lgy* infection promoting parasite survival by controlling the extent of the oxidative burst in infected cells. Taken together, these data confirmed that activation of NRF2 occurred with every *L*. *spp*. tested leading to the activation of downstream genes and ROS control and did not depend on LRV1 nor on TLR signaling at least with the *L*. *spp*. tested.

### NRF2 participates to lesion development in WT mice and controls disease severity in immunocompromised mice infected with *Lgy*LRV1+

**Fig 2:**
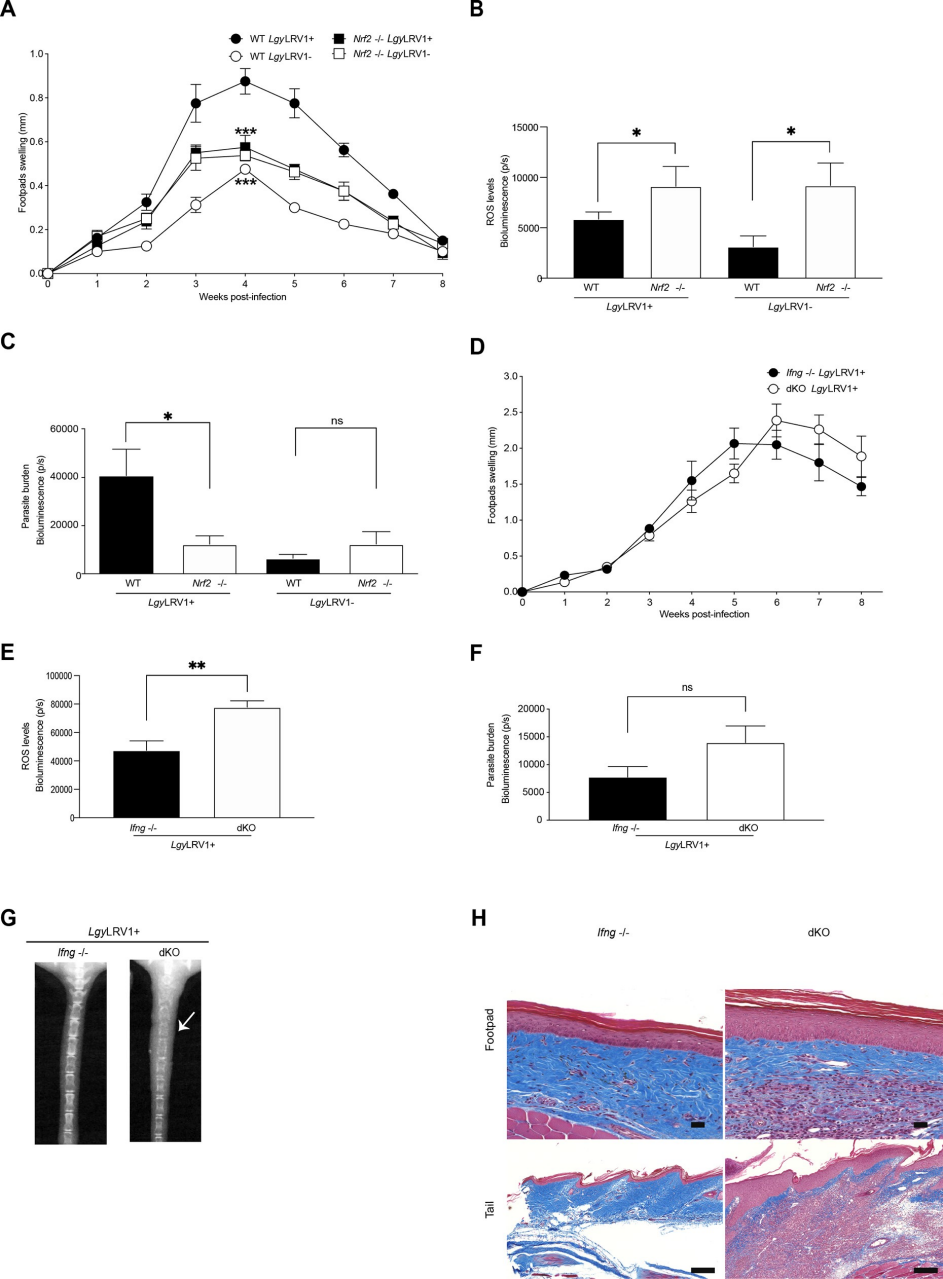
NRF2 participates to lesion development in WT mice and controls disease severity in immunocompromised mice infected with *Lgy*LRV1+. WT, *Nrf2*^-/-^, *Ifng*^-/-^ or *Ifng*x*Nrf2* dKO (dKO) mice were infected in both hind footpads with 1x10^6^ of either *Lgy*LRV1+ or *Lgy*LRV1- parasites containing the bioluminescent luciferase gene. A) and D) Footpad swelling evolution was measured weekly as a proxy for disease progression for WT and *Nrf2*^-/-^ mice (A) and *Ifng*^-/-^ and dKO mice (D). B and E) ROS levels were measured by luminol-generated bioluminescence at week 4 for WT and *Nrf2* ^-/-^ mice (B) or at week 5 for *Ifng* ^-/-^ and dKO mice (E). C and F) Parasite burden was quantified by luciferin detecting parasite bioluminescence either at week 4 for WT and *Nrf2*^-/-^ mice (C) or at week 5 for *Ifng*^-/-^ and dKO mice (F). G) Representative X-Ray images indicating tail tissue and bone destruction at week 8 for *Ifng*^-/-^ and dKO mice. H) Representative images of Masson’s trichrome staining of footpad and tail sections at week 8 for *Ifng*^-/-^ and dKO mice at 20x on an automated slide scanning microscope. Collagen (blue), nuclei (black), muscle and cytoplasm (red) are represented. Scale bar represents 50 μm or 200 μm for footpad and tail images, respectively. *In vivo* bioluminescence measurements were performed using Xtreme II (BRUKER) imaging system. Data represents the mean ± SEM or representative images from one representative experiment (n = 3–5 mice) of three independent experiments. Statistical significance was assessed by two-way ANOVA analysis with Bonferroni’s post-test (A, D, E and F) and unpaired Student’s t test (B and C). Not significant (ns), * p < 0.05, ** p < 0.01 and *** p < 0.001. See also S2 Fig.

To evaluate the role of NRF2 in the modulation of *Lgy* pathogenicity *in vivo*, we infected WT and *Nrf2*^-/-^ mice. We observed a drastic decrease in footpad swelling at the peak of infection in *Nrf2*^-/-^ mice infected with *Lgy*LRV1+ in comparison to WT mice but not in *Lgy*LRV1- infection (Fig 2A). This suggested that NRF2 played a similar protective role in *in vitro* infected macrophages for both *Lgy*LRV1+ and *Lgy*LRV1- (Fig 1F) but only for *Lgy*LRV1+ in mice (Fig 2A). Consistent with the ROS findings in macrophages (Fig 1D), *Nrf2*^-/-^ mice presented higher levels of ROS with both *Lgy*LRV1+ and *Lgy*LRV1- at the peak of infection in comparison to their WT counterparts (Fig 2B), using bioluminescence *in vivo* imaging of footpads, which suggested also no difference in ROS control in between *Lgy*LRV1+ and *Lgy*LRV1- infected mice. On the other hand, using bioluminescence *in vivo* imaging of footpads infected with *Lgy*LRV1+ and *Lgy*LRV1- expressing luciferase [56], we found that *Nrf2*^-/-^ mice infected with *Lgy*LRV1+, but not with *Lgy*LRV1-, displayed a significant decrease in parasite numbers in comparison to their WT counterparts (Fig 2C). Altogether, these findings suggested that NRF2 activation controlled ROS in a similar manner between *Lgy*LRV1+ and *Lgy*LRV1- but its expression was beneficial for the survival of *Lgy*LRV1+ parasites (Fig 2C). As expected in *Nrf2*^-/-^ mice, we observed a significant decrease in the expression of *Hmox1* and *Nqo1*, two target genes implicated in the antioxidant response (S2A and S2B Fig). Considering that in *Lam* infection, *Hmox1* was implicated in parasite survival [41], we decided to verify whether HO-1 could play a similar role as NRF2 in *Lgy*LRV1+ infection. To investigate this, we used conditional knock-out (KO) mice, as *Hmox1*^-/-^ full KO are not viable [57], and infected them with *Lgy*LRV1+ parasites. No difference could be observed between the control mice, *Hmox1*fl/flxLysm^+/-^, or *Hmox1*fl/flxLysm^-/-^ (S2C Fig). Thus, we excluded a possible role of *Hmox1* in mice infected with *Lgy*LRV1+. Taken together, these data pointed to a possible role of NRF2 on the control of inflammatory cytokines, such as IFN-γ and TNF-α, which act in concert for an effective immune response against *Leishmania* or play a detrimental role in inflammatory leishmaniasis such as MCL [6,58].

An oxidative environment such as in *Nrf2*^-/-^ mice leads to an increased sensitivity to the proinflammatory response [46,50], we thus decided to measure TNF-α, as a proxy of the activation of NF-κB-dependent pathway by the LRV1/TLR-3 axis. We analyzed the transcripts of *Tnfa* gene in the footpads of WT and *Nrf2*^-/-^ mice infected with *Lgy*LRV1+ at 3 weeks post-infection. As shown (S2D Fig), we detected an increase in *Tnfa* mRNA expression in footpads of *Nrf2*^-/-^ mice infected with *Lgy*LRV1+ in comparison to WT mice. This result supported the assumption that NRF2 participated in the control of the expression level of *Tnfa* in *Leishmania* infection.

We decided to further investigate the role of NRF2 in hyperinflammatory situations exemplified by *Lgy*LRV1+ infections by infecting *Ifng*^-/-^ mice, which exhibit exacerbated disease outcome, hyperinflammation and IL-17-dependent metastatic lesions [10]. We therefore evaluated the impact of NRF2 in the absence of IFN-γ by crossing *Nrf2*^-/-^ mice with *Ifng*^-/-^ mice to generate *Ifng*x*Nrf2* double-knockout (dKO) mice. *Ifng*^-/-^ and dKO mice were infected with *Lgy*LRV1+ parasites. Our data showed that lesion size of both *Ifng*^-/-^ and dKO mice were similar suggesting that absence of NRF2 did not limit lesion development in an *Ifng*^-/-^ environment (Fig 2D) contrary to WT mice. The absence of difference in footpad size of *Ifng*^-/-^ and dKO mice was not surprising and can be explained by the absence of IFN-γ, which normally acts with TNF-α in killing of intracellular *Leishmania* parasites [52].

As observed in *Nrf2*^-/-^ mice, dKO mice presented greater ROS levels at week 6 post-infection (Fig 2E) and less expression of NRF2-target genes *Hmox1* and *Nqo1* as measured at week 3 post-infection on total footpad lesions caused by *Lgy*LRV1+ parasites (S2E and S2F Fig). Comparable parasite burden levels were detected between dKO mice and their *Ifng*^-/-^ counterparts at week 6 post-infection, as measured by bioluminescence of luciferase expressing parasites (Fig 2F). This showed that, in the absence of IFN-γ, NRF2 did not influence the development of lesions in *Lgy*LRV1+ infected mice but its absence influenced inflammation by increasing TNF-α. Similar levels of *Tnfa* gene were observed at week 3 post-infection between *Ifng*^-/-^ and dKO mice (S2G Fig). However, enhanced transcripts of the *Tnfa* gene were detected in the footpads and tails of dKO mice at the end of the infection (S2H and S2I Fig). As expected, we did not observe any difference in cartilage destruction of the tail between WT and *Nrf2* ^-/-^ mice (S2J Fig) since dissemination of the infection to secondary sites is not expected to occur in the presence of IFN-γ [10]. Similarly, we did not observe any significant difference in the footpad skin biopsies of WT and *Nrf2* ^-/-^ mice at 3 weeks post-infection (S2K Fig). In contrast, we observed that dKO mice displayed higher cartilage destruction in the tail region compared to *Ifng*^-/-^ as visualized by X-Ray imaging (Fig 2G), which supported a role of NRF2 as limiting inflammation and preventing excess pathology. Concomitantly, we observed an increased epidermal hyperplasia and dermal infiltration by immune cells in footpads of dKO mice at the end of the infection (Fig 2H), but no significant differences were observed at 3 weeks post-infection between *Ifng*^-/-^ and dKO mice (S2K Fig). Therefore, in the dKO model, our results provided evidence that NRF2 expression was beneficial for the host to prevent tissue destruction and disease severity during *Lgy*LRV1+ infection in metastatic mice likely by controlling NF-κB and limiting inflammation but also to the parasite by limiting TNF-α and its anti-*Leishmania* activity.

### NRF2 expression controls inflammation in *Lgy*LRV1+ infected macrophages

**Fig 3:**
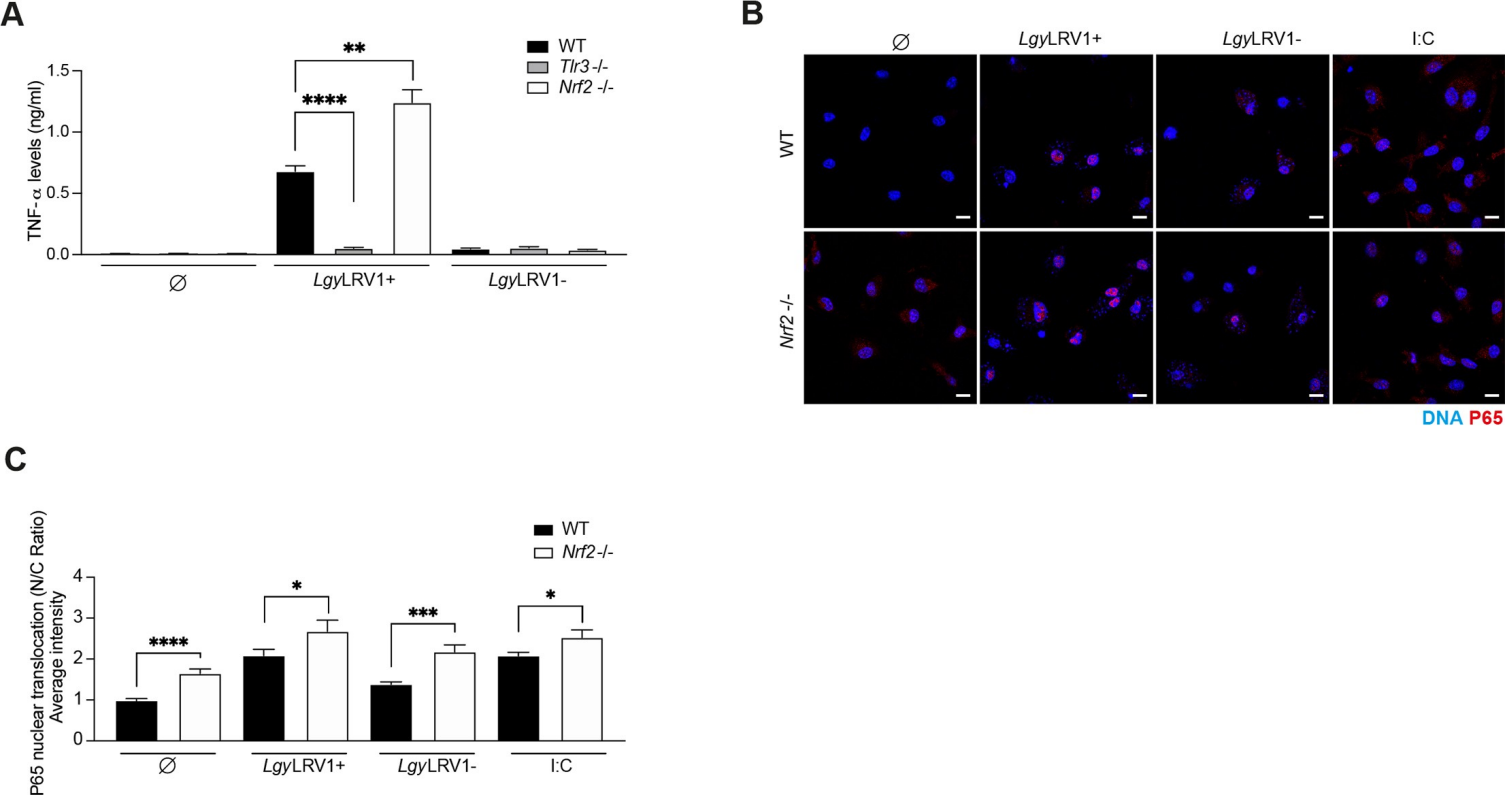
NRF2 expression controls inflammation in *Lgy*LRV1+ infected macrophages. WT, *Tlr3*^-/-^ and *Nrf2*^-/-^, BMDMs were infected with either *Lgy*LRV1+ or *Lgy*LRV1- parasites. Negative and positive controls of non-treated (Ø), tBHQ (10 μM), poly I:C (I:C, 2 μg/ml), TLR3 agonist, were performed concurrently. A) Secreted levels of TNF-α into the supernatant at 24 hr time-point by ELISA. B) Immunofluorescence of nuclear translocation of P65 subunit of NF-κB complex expressed as nucleus/cytoplasm (N/C) ratio. Cells were stained with NRF2 (red), DNA (blue) at 24 hrs and imaged at 63x using a confocal microscope. Scale bar represents 10 μm. C) Quantification of P65 nuclear translocation at 24 hrs. P65 nuclear translocation was quantified for each cell (N = 50–90) by IMARIS software. Representative images from two independent experiments are shown. Data show mean ± SEM from a pool of three (A) and two (C) independent experiments. Unpaired Student’s t test was used to assess statistical significance. Not significant (ns), * p < 0.05, ** p < 0.01, *** p < 0.001, **** p < 0.0001.

To further test whether NRF2 was involved in controlling inflammation, we measured TNF-α cytokine in the supernatant of macrophages infected with *Lgy* parasites using ELISA. We observed that higher levels of TNF-α were detected in *Nrf2*^-/-^ macrophages infected with *Lgy*LRV1+ (Fig 3A), but not in the supernatant of macrophages infected with *Lgy*LRV1-. As expected for parasites harboring LRV1dsRNA, TNF-α production was TLR-3 dependent (Fig 3A). Thus, TNF-α expression was depending on LRV1 acting on TLR-3, but its level was controlled by NRF2 possibly by modulating the NF-κB pathway. To substantiate the interplay between NRF2 and the NF-κB pathway in *Lgy* infection, we visualized and quantified nuclear translocation of P65 subunit of NF-κB complex in WT and *Nrf2*^-/-^ BMDMs using confocal microscopy and IMARIS software, respectively at 24 hrs. *Nrf2*^-/-^ cells presented higher levels of nuclear P65 at basal level without stimulation, in *Lgy*LRV1+ and *Lgy*LRV1- infected cells or in poly (I:C) treated cells used as positive control for LRV1 effect (Fig 3B and 3C). We also measured the transcripts of the *Nfkb1* gene in WT and *Nrf2*^-/-^ cells infected with *Lgy* at 8 hrs, which encodes for the p105 protein that serves as a precursor of p50 subunit of the p65/p50 canonical NF-κB complex. Our results revealed enhanced expression of the *Nfkb1* gene in *Nrf2*^-/-^ cells infected with *Lgy*LRV1+ parasites (S3 Fig). Taken together, this data suggested that activation of the NRF2 pathway in *Lgy*LRV1+ infection resulted in the control of the NF-κB pathway and thereby limited the detrimental effect of LRV1/TLR-3 axis.

### NOX2 promotes NRF2 signaling through ROS in *Lgy*LRV1+ and *Lgy*LRV1- infections

**Fig 4:**
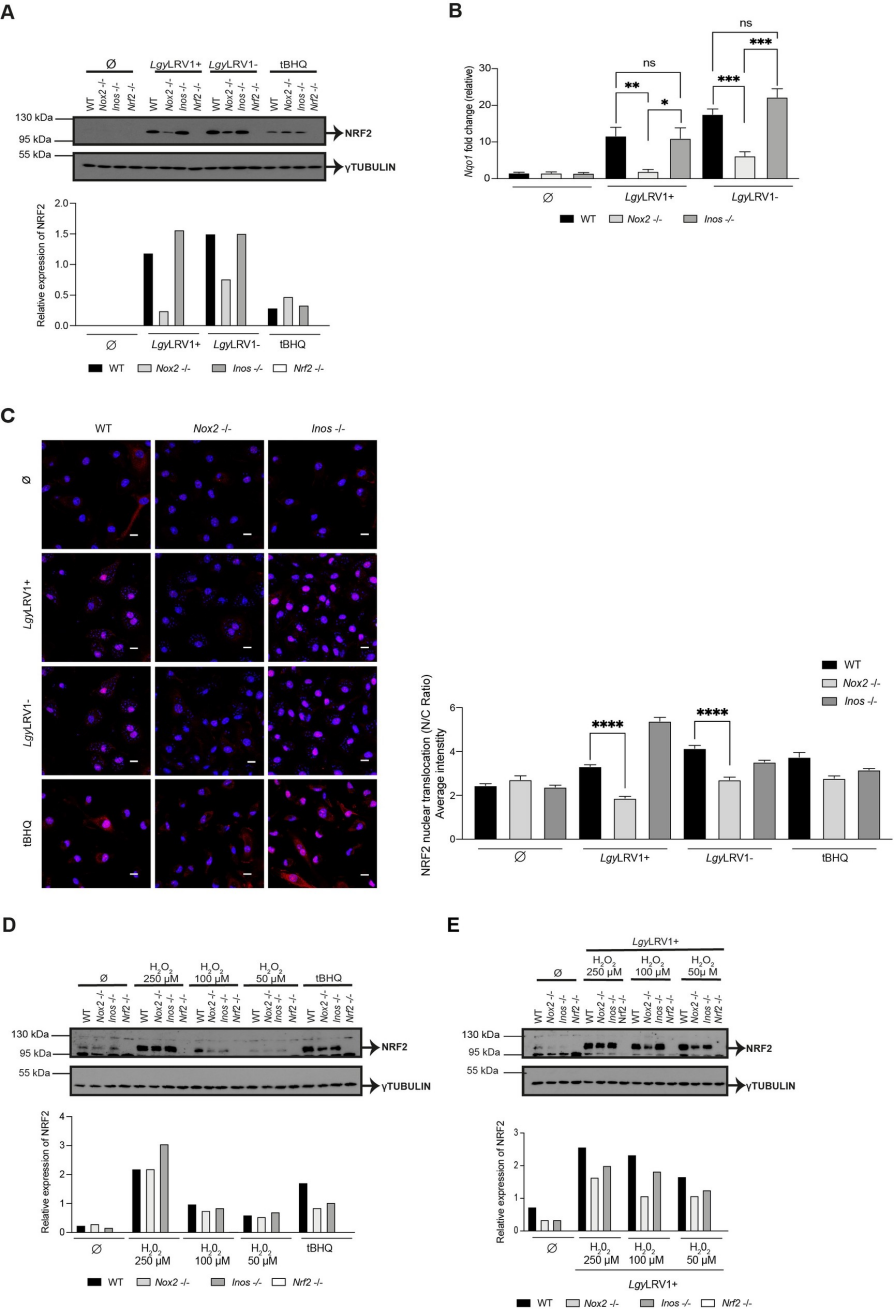
NOX2 promotes NRF2 signaling through ROS in *Lgy*LRV1+ and *Lgy*LRV1- infections. BMDMs from WT, *Nox2*^-/-^, *Inos*^-/-^ and *Nrf2*^-/-^ mice were either non-stimulated (Ø), or infected with *Lgy*LRV1+ or *Lgy*LRV1- parasites, or stimulated with tBHQ (10 μM). A) Cells lysates were collected at 4 hrs post-infection and NRF2 protein levels were assessed by Western Blot using anti-NRF2 and anti-γTUBULIN antibodies. Relative NRF2 levels were determined by band quantification using Image J software and given as NRF2 over γTUBULIN. B) *Nqo1* relative RNA expression levels measured at 8 hrs post-infection by RT-qPCR and normalized to *L32* housekeeping gene. C) Immunofluorescence and quantification of NRF2 nuclear translocation expressed as nucleus/cytoplasm (N/C) ratio in WT, *Nox2*^-/-^ and *Inos*^-/-^ BMDMs. Cells were stained with NRF2 (red), DNA (blue) at 8 hrs and imaged at 63x using a confocal microscope. Scale bar represents 10 μm. NRF2 nuclear translocation was quantified for each cell (N = 50–160) by IMARIS software. D and E) Cells were cultured with medium only (Ø) (D) or infected with *Lgy*LRV1+ (E) for 6 hrs and treated with three different concentrations (250 μM, 100 μM, 50 μM) of the ROS donor, H_2_O_2_ for 2 hrs. Cell lysates were analyzed by Western blot for assessing NRF2 protein expression, γTUBULIN served as positive control. Relative NRF2 levels were determined by band quantification using Image J software and given as NRF2 over γTUBULIN. Representative blots and images and their quantification from three independent experiments. Data is expressed as mean ± SEM (B) from a pool of three independent experiments. Significance was calculated by Student’s t test. Not significant (ns), * p < 0.05, ** p < 0.01, *** p < 0.001, **** p < 0.0001. See also S4 Fig.

Knowing that NRF2 activation occurred with both *Lgy*LRV1+ and *Lgy*LRV1- and was independent of TLRs (S1F and S1G Fig), we focused on a general mechanism implicating ROS and/or RNS, which can be produced in macrophages by NADPH oxidase 2 (NOX2) and inducible nitric oxide synthase (iNOS), respectively [59]. We thus tested whether ROS or RNS were responsible for NRF2 pathway activation in *Lgy* infection by using NOX2 (*Nox2*^-/-^) and iNOS (*Inos*^-/-^) deficient mice. NRF2 protein levels were evaluated in WT, *Nox2*^-/-^, *Inos*^-/-^ and *Nrf2* ^-/-^ macrophage lysates at 4 and 8 hrs post-infection. Lower levels of NRF2 in *Nox2* ^-/-^ cells were observed in comparison to WT and *Inos*^-/-^ cells at both time-points (Figs 4A and S4A). Moreover, NOX2 modulated NRF2 protein levels in *Lgy* independently of LRV1 as no notable difference between *Lgy*LRV1+ and *Lgy*LRV1- could be detected (Figs 4A and S4A). Interestingly, the absence of NOX2 had an impact on the expression of NRF2 at the RNA level as measured by qRT-PCR (S4B Fig). Additionally, the level of *Nqo1* gene was also significantly reduced in *Nox2*^-/-^ macrophages infected with *Lgy*LRV1+ and *Lgy*LRV1- but not in WT and *Inos*^-/-^ cells at 8 hrs post-infection (Fig 4B). By using confocal microscopy at an 8 hr time-point, we could correlate these findings with significantly decreased ratios of nuclear to cytoplasmic NRF2 in *Nox2*^-/-^ cells in comparison to WT cells in both *Lgy*LRV1+ and *Lgy*LRV1- infections (Fig 4C). We finally tested whether NRF2 expression in macrophages was dependent on NOX2 between the different *L*. *spp*. Our data indicated that NOX2-dependent NRF2 activation was conserved along the different *L*. *spp*. tested at 4 hrs post-infection (S4C and S4D Fig).

To validate NRF2 activation by an oxidative burst produced by NOX2 in *Lgy* infection, we used H_2_O_2_, as ROS donor. WT, *Nox2*^-/-^, *Inos*^-/-^ and *Nrf2* ^-/-^ BMDMs were incubated with different doses of H_2_O_2_ in the absence and in the presence of *Lgy*LRV1+ parasites and the NRF2 expression level was evaluated at an 8 hr time-point (Fig 4D and 4E). It was observed that a high concentration of H_2_O_2_ induced NRF2 expression in WT, *Nox2*^-/-^ and *Inos*^-/-^ cells or in *Nox2*^-/-^ cells infected with *Lgy*LRV1+ complementing NOX2 deficiency (Fig 4D and 4E). Taken together, all these results provided evidence that NRF2 induction in *Leishmania* infection was dependent on NOX2/ROS modulation.

### Decreasing phagocytosis did not block NRF2 activation in *Lgy* infection

**Fig 5:**
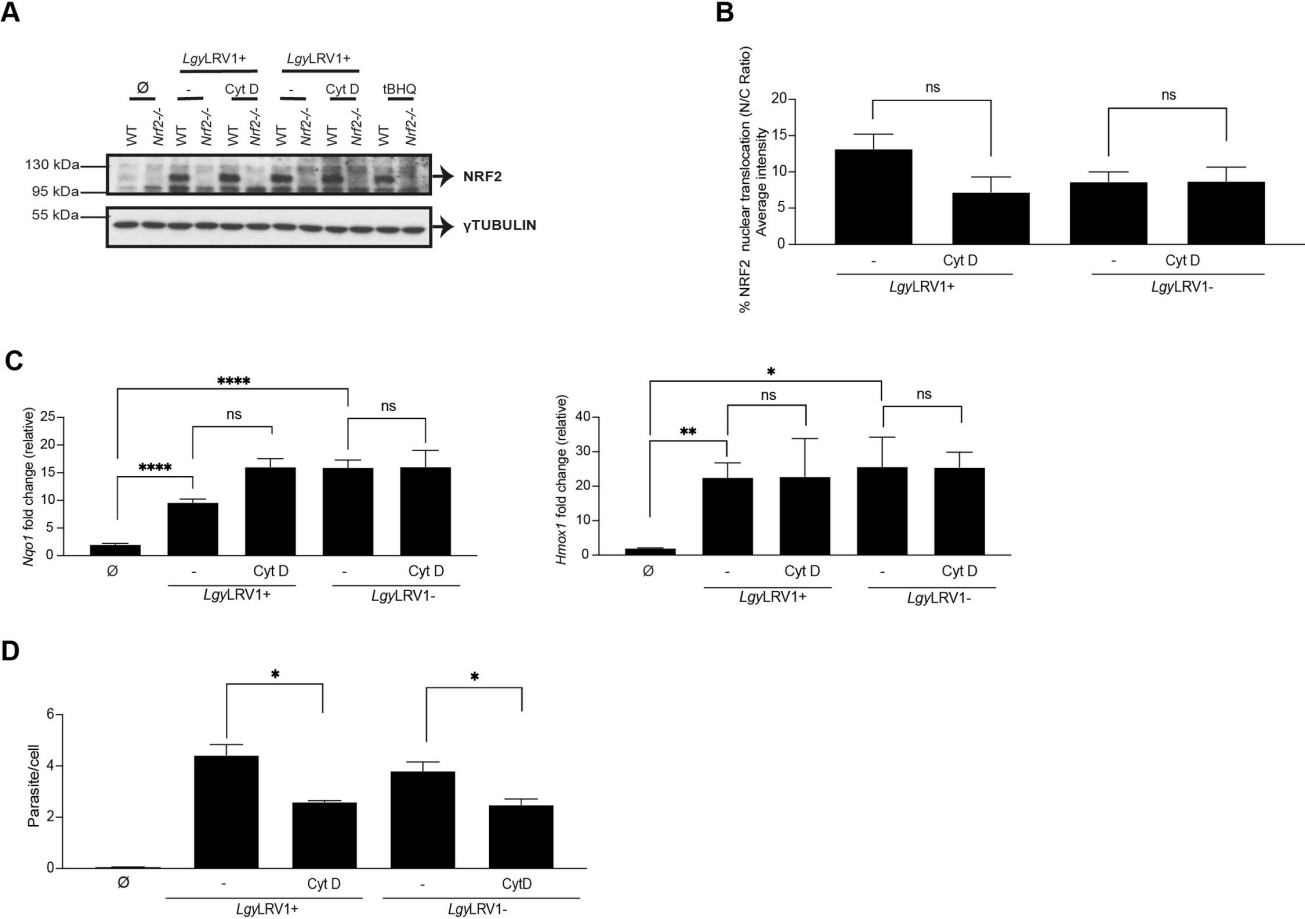
Decreasing phagocytosis did not block NRF2 activation in *Lgy* infection. A-D) WT, *Nox2*^-/-^ or *Nrf2*^-/-^ cells were pretreated with DMSO or Cytochalasin D (Cyt D, 40 μM) for 1 hr and infected with *Lgy*LRV1+ or *Lgy*LRV1- parasites or stimulated with tBHQ (10 μM), or medium-treated (Ø). A) Cells lysates were immunoblotted with anti-NRF2 and anti-γTUBULIN antibodies. B) WT cells were stained with NRF2 and DAPI for assessing NRF2 nuclear translocation expressed as nucleus/cytoplasm (N/C) ratio at 8 hrs at 40x using a high-content microscope. NRF2 nuclear translocation was quantified using MetaXpress software and normalized to non-infected (Ø). C) *Nqo1* and *Hmox1* relative RNA levels were measured by RT-qPCR at 8 hrs post-infection and normalized to *L32* housekeeping gene. D) Intracellular parasite load was quantified by DAPI staining at 8 hrs at 40x using a high-content microscope. Parasite load was quantified using MetaXpress software. Representative blots are shown from three independent experiments. The graphs show pooled data expressed as mean ± SEM from two independent experiments. Unpaired Student’s t test was used to measure statistical significance. Not significant (ns), * p < 0.05, ** p < 0.01 and **** p < 0.0001. See also S5 Fig.

We decided to investigate if parasite phagocytosis was involved in NRF2 activation through the NOX2 protein by pretreating the cells with cytochalasin D, an inhibitor of phagocytosis that prevents actin polymerization [60]. WT, *Nox2*^-/-^ and *Nrf2*^-/-^ BMDMs were pretreated with cytochalasin D and subsequently infected with either *Lgy*LRV1+ or *Lgy*LRV1- parasites or treated with tBHQ for 4 and 8 hrs. Altering phagocytosis did not affect NRF2 expression (Figs 5A and S5A) nor its nuclear translocation in *Lgy* infected cells (Fig 5B). Concomitantly, we detected that *Nqo1* and *Hmox1* gene expression were not affected by cytochalasin D pretreatment in *Lgy* infection (Fig 5C). However, we could observe a decrease in the number of intracellular parasites (Fig 5D). We confirmed cytochalasin D efficacy since we observed an impaired proinflammatory response mediated by *Lgy*LRV1+ parasites (S5B Fig), as previously reported [8]. Our findings revealed that NRF2 activation might not depend exclusively on phagocytosis of parasites.

### Initial contact of *Leishmania* with its host cell activates the NRF2 pathway

**Fig 6:**
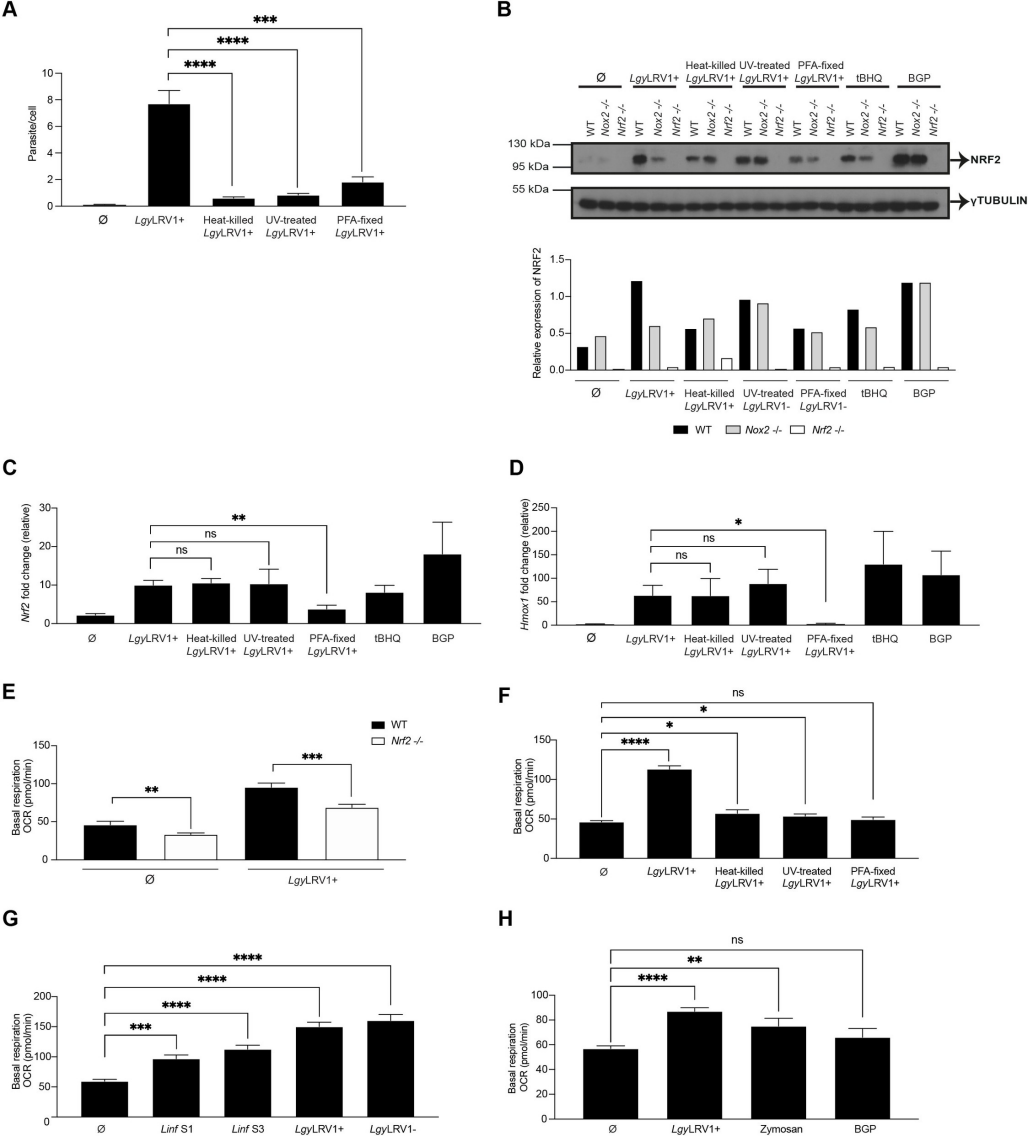
Initial contact of *Leishmania* with its host cell activated the NRF2 pathway and parasite entry strongly induced metabolic reprogramming. A) WT cells were infected with live, or heat-killed, or UV-treated or PFA-fixed *Lgy*LRV1+ parasites, or were non-infected (Ø) for 8 hrs. Intracellular parasite load was quantified by DAPI staining at 40x using a high-content microscope. Parasite load was quantified using MetaXpress software. B) WT, *Nox2*^-/-^ or *Nrf2*^-/-^ cells were either infected with live or heat-killed or UV-treated or PFA-fixed *Lgy*LRV1+ parasites. Non-treated (Ø) or tBHQ-treated (10 μM), or β-glucan peptide (BGP)-treated (100 *μ*g/ml) cells were performed concurrently. Cell lysates after 4 hrs post-infection were analyzed by Western Blot using anti-NRF2 and anti-γTUBULIN antibodies. Relative NRF2 levels were determined by band quantification using Image J software and given as NRF2 over γTUBULIN. C and D) WT cells were either infected with live, or heat-killed, or UV-treated or PFA-fixed *Lgy*LRV1+ parasites, or non-infected (Ø) for 8 hrs. *Nrf2* (C) or *Hmox1* (D) relative RNA levels were measured by RT-qPCR and normalized to the *L32* housekeeping gene. E) WT and *Nrf2*^-/-^ cells *Lgy*LRV1+ parasites or non-infected (Ø) for 15 min and basal respiration represented as OCR was measured on Seahorse XFe96 Analyzer, adjusted to protein concentration per condition. F) WT cells were either infected with live, or heat-killed, or UV-treated or PFA-fixed *Lgy*LRV1+ parasites, or non-infected (Ø) for 15 min and basal respiration represented as OCR was measured on Seahorse XFe96 Analyzer, adjusted to protein concentration per condition. G) WT cells were either infected with *Linf* S1 or *Linf* S3 or *Lgy*LRV1+ or *Lgy*LRV1- parasites, or non-infected (Ø) for 15 min and basal respiration represented as OCR was measured on Seahorse XFe96 Analyzer, adjusted to protein concentration per condition. H) WT cells were infected with *Lgy*LRV1+ parasites, or non-infected (Ø) zymosan particles-treated (1 *μ*g/ml) or β-glucan peptide (BGP)-treated (100 *μ*g/ml) cells for 15 min and basal respiration represented as OCR was assessed on Seahorse XFe96 Analyzer, adjusted to protein concentration per condition. Representative blots and their quantification from two independent experiments. The graphs show pooled data expressed as mean ± SEM from three independent experiments. Unpaired Student’s t test was used to measure statistical significance. Not significant (ns), * p < 0.05, ** p < 0.01, *** p < 0.001 and **** p < 0.0001. See also S6 Fig.

Considering that phagocytosis was possibly not essential for NRF2 activation (Figs 5 and S5), we investigated different treatments which could affect entry of parasites into macrophages. We treated *Lgy*LRV1+ parasites with UV, 95°C heat or fixed them with PFA and incubated macrophages with treated parasites for different time-points. Heat-killed, UV-treated or PFA-fixed parasites did not enter macrophages in comparison to non-treated *Lgy*LRV1+ at 8 hrs post-infection (Fig 6A). We then analyzed whether treated *Leishmania* parasites activated the NRF2 pathway. We compared NRF2 expression at a 4 hr time-point in WT, *Nox2*^-/-^ and *Nrf2*^-/-^ macrophages infected with live *Lgy*LRV1+ or killed by PFA fixation, heat or UV treatment, or treated with tBHQ or BGP (β-glucan peptide), a fungal glucose polymer agonist of Dectin-1/TLR-2 receptor that triggers phagocytosis, respiratory burst and inflammatory responses [61]. Our results showed that in WT but not in *Nox2*^-/-^ cells NRF2 was increased in all conditions except when exposed to PFA-fixed *Lgy*LRV1+ (Figs 6B, S6A and S6B). In this latter case, NRF2 was not, or only weakly induced (Figs 6B and S6A). No effect on NRF2 protein expression was also observed in other PFA-fixed *L*.*spp*. (S6C Fig). Similarly, *Nrf2* gene and NRF2-target gene expression were not induced in *Lgy*LRV1+ PFA-fixed parasites in comparison to live, UV- or heat-treated *Lgy*LRV1+ at 8 hrs post-infection of WT cells (Figs 6C, 6D and S6D). These results indicated that PFA fixation, which alters parasite surface molecules, prevented parasite recognition by macrophages. In addition, PFA-fixed and heat-killed *Lgy*LRV1+ did not induce the pro-inflammatory response since only very low levels of TNF-α were detected at 24 hrs post-infection by ELISA in comparison to live or UV-treated *Lgy*LRV1+ (S6E Fig) suggesting that heat and PFA treatment of parasites possibly prevented the activation of the LRV1/TLR-3 axis. Thus, initial *Leishmania* contact was necessary and sufficient to promote macrophage activation resulting in upregulation of the NRF2 pathway response via NOX2. Interestingly, NOX2 dependency was not observed when macrophages were treated with heat-killed, UV-treated or PFA fixed parasites, tBHQ or BGP, conditions where there was no phagocytosis.

Mitochondrial metabolism has been reported to be upregulated by NRF2 activation since *Nrf2*^-/-^ cells present decreased mitochondrial oxidative phosphorylation (OXPHOS) [62,63] and previous studies reported that *Leishmania* infection increases OXPHOS in macrophages [64]. To further investigate the importance of the parasite cell surface in the activation of NRF2, we assessed if there was a difference in the metabolic reprogramming response of macrophages exposed to heat-killed, UV-treated, or PFA-fixed *Lgy*LRV1+ parasites. We then determined the mitochondrial oxygen consumption rate (OCR) in non-infected or *Lgy*LRV1+ infected WT and *Nrf2*^-/-^ cells. We showed that OCR was already decreased in *Nrf2*^-/-^ cells compared to WT cells (Fig 6E) but was higher in macrophages incubated with *Lgy*LRV1+ than when incubated with heat-killed or UV-treated, or PFA-fixed *Lgy*LRV1+ parasites for only 15 min using Seahorse XFp respirometer (Fig 6F). Basal respiration, which shows the energetic demand of the cell under baseline conditions, indicated that *Nrf2*^-/-^ cells presented lower OCR in comparison to WT cells and only live *Lgy*LRV1+ augmented the cell metabolism (Fig 6E). PFA-fixed *Lgy*LRV1+ parasites did not significantly induce the metabolic reprogramming response of macrophages whereas heat-killed or UV-treated parasites did slightly (Fig 6F). It confirmed that parasite cell surface contact was important to activate the NRF2 pathway, and that parasite internalization was required to efficiently induce the metabolic switch of the cell in *Lgy* infection. Macrophage metabolic reprogramming was also tested with two strains of *Linf* and with *Lgy*LRV1+ and *Lgy*LRV1- parasites in infected WT cells for 15 min. Similarly, to *Lgy*, both *Linf* strains significantly induced changes in the cell metabolism (Fig 6G). These preliminary data suggested that infection and entry of *Leishmania* were important to efficiently induce changes in metabolism. It could be interesting to further validate these results with other *L*. *spp*.

Finally, to verify that phagocytosis was required to induce the cell metabolic reprograming dependent on NRF2, we infected WT macrophages with live *Lgy*LRV1+ parasites, or treated them with zymosan particles, or BGP for 15 min. Zymosan particles consist of a cell wall preparation rich in β-glucan that activates macrophages via Dectin-1/TLR-2 inducing inflammatory signals. Phagocytic receptors of macrophages internalize zymosan particles upon their recognition [65], contrary to BGP, which is not phagocytized. Our results showed that basal respiration of zymosan particles increased OCR as observed with *Lgy*LRV1+ parasites (Fig 6H). BGP, in turn, showed basal respiration levels similar to non-treated conditions (Fig 6H). Thus, although both zymosan particles and BGP induced NRF2 protein expression (Figs 6B and S6F) at the 4 hr time-point, only phagocytized zymosan particles significantly promoted oxidative metabolism (Fig 6H). These data indicated that host contact was not sufficient to induce the cell metabolic reprogramming which required phagocytosis supporting that 15 min infection is sufficient to induce parasite phagocytosis as previously described [14] and we confirmed parasite internalization at this short time-point (S6G Fig). Collectively, these data suggested that NRF2 activation in *Leishmania* parasites was induced by cell surface contact, but cell metabolic reprogramming required phagocytosis.

### *Leishmania* activates the host cell NRF2 pathway through a SRC family kinase signaling cascade

**Fig 7:**
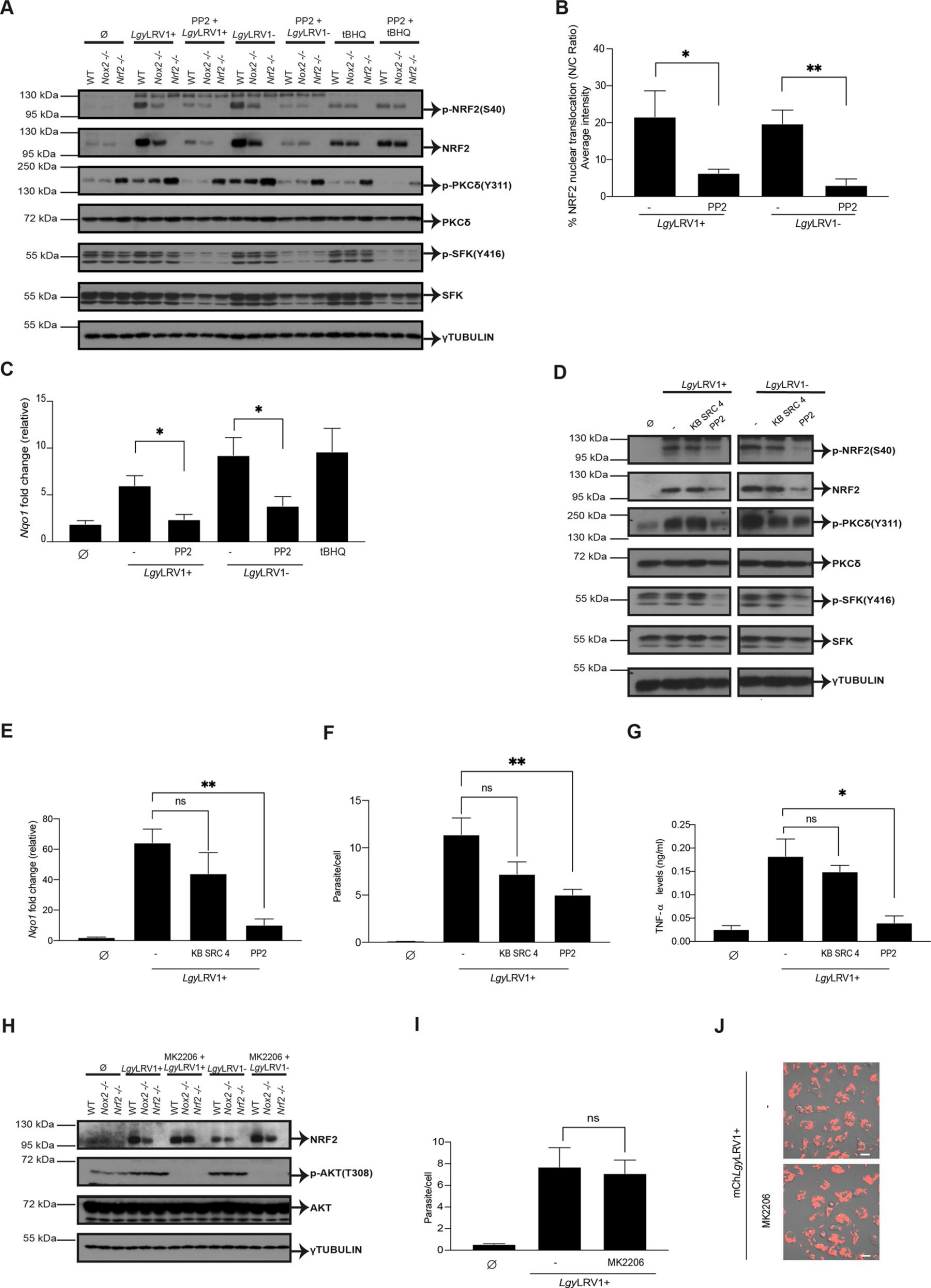
*Leishmania* activates the host cell NRF2 pathway through an SRC family kinase signaling cascade. A-C) WT, *Nox2*^-/-^ or *Nrf2*^-/-^ cells were pretreated with DMSO or PP2 (100 μM) for 1 hr and infected with *Lgy*LRV1+ or *Lgy*LRV1- parasites. Non-treated (Ø) or tBHQ-treated (10 μM) cells were performed concurrently. A) Cell lysates after 4 hrs post-infection were analyzed by Western Blot using anti-phosho-NRF2 (S40), anti-NRF2, anti-phospho-PKCδ (S311), anti-PKCδ, anti-phospho-SFK (Y416), anti-SFK and anti-γTUBULIN antibodies. B) Cells were stained with NRF2 and DAPI for assessing NRF2 nuclear translocation expressed as nucleus/cytoplasm (C/N) ratio at 8 hrs with a 40x using a high-content microscope. NRF2 nuclear translocation was quantified using MetaXpress software and normalized to non-infected (Ø). C) *Nqo1* relative RNA levels were measured by RT-qPCR and normalized to *L32* housekeeping gene. D) WT cells were pretreated with DMSO (-) or PP2 (100 μM) or KB SRC 4 (100 μM) for 1 hr and infected with *Lgy*LRV1+ or *Lgy*LRV1- parasites. Non-treated (Ø) cells were performed concurrently. Cell lysates after 4 hrs post-infection were analyzed by Western Blot using anti-phosho-NRF2 (S40), anti-NRF2, anti-phospho-PKCδ (S311), anti-PKCδ, anti-phospho-SFK (Y416), anti-SFK and anti-γTUBULIN antibodies. E-G) WT cells were pretreated with DMSO (-) or PP2 (100 μM) or KB SRC 4 (100 μM) for 1 hr and infected with *Lgy*LRV1+. E) *Nqo1* relative RNA levels were measured at an 8 hr time-point by RT-qPCR and normalized to *L32* housekeeping gene. F) Intracellular parasite load was quantified by DAPI staining at 40x using a high-content microscope at 8 hrs post-infection. Parasite load quantified using MetaXpress software. G) Secreted levels of TNF-α into the supernatant at 24 hr time-point by ELISA. H-J) WT, *Nox2*^-/-^ or *Nrf2*^-/-^ cells were pretreated with DMSO or MK2206 (5 μM) for 1 hr and infected with either *Lgy*LRV1+ or *Lgy*LRV1- parasites, or non-treated (Ø). H) Cell lysates after 4 hrs post-infection were analyzed by Western Blot using anti-NRF2, anti-phospho-AKT (T308), anti-AKT and anti-γTUBULIN antibodies. I) Intracellular parasite load was quantified by DAPI staining at 40x using a high-content microscope at 8 hrs post-infection. Parasite load quantified using MetaXpress software. H) Representative image of live cell imaging performed to visualize parasite internalization at 20x by confocal microscopy using mCh*Lgy*LRV1+ (red) parasites. Scale bar: 40 μm. Anti-SFK and anti-phospho-SFK antibodies recognize SFK members including Lyn, Fyn, Lck, Yes and Hck proteins and their corresponding phosphorylation sites. Representative blots and images from two (D and I) and three (A) independent experiments are shown. The graphs show pool data expressed as mean ± SEM from two independent experiments. Unpaired Student’s t test was used to calculate statistical significance. Not significant (ns), * p < 0.05 and ** p < 0.01. See also S7 Fig.

Members of the SFKs can function as redox sensors [66]. In particular, oxidative stress has been described to activate SRC subfamily C kinase (c-SRC) [67–69] and c-SRC has also been reported to play a role in NRF2 activation. Since phosphorylation of c-SRC activates the phosphorylation of the PKCδ isoform, which in turn phosphorylates NRF2 at serine 40 residue (S40) [27,29], we tested whether NOX2/ROS could activate the NRF2 pathway in *Leishmania* infection by activating a SFK /PKCδ axis. To study the role of SFKs in NRF2 activation in *Lgy* infection, we used the phosphorylation level of Y416 as a proxy of SFKs catalytic activity and PP2, a selective inhibitor for SFKs. In particular, PP2 efficiently inhibits mainly three members of the SFKs (Fyn, Hck and Lck) [70]. WT, *Nox2*^-/-^ and *Nrf2*^-/-^ BMDMs were pretreated for 1 hr with PP2 and subsequently infected with either *Lgy*LRV1+ or *Lgy*LRV1- parasites, or treated with tBHQ for 4 and 8 hrs. We observed that PP2 pretreatment resulted in decreased phosphorylation levels of NRF2 (S40), PKCδ (Y311) and SFK (Y416) proteins in both *Lgy*LRV1+ and *Lgy*LRV1- infection, in both WT and *Nox2*^-/-^ cells at the two time points (Figs 7A and S7A). NRF2 activation was not affected by PP2 pretreatment when stimulating the cells with tBHQ (Figs 7A and S7A) suggesting that the role of the SFKs was associated with *Leishmania* infections and possibly NOX2. Interestingly, *Nrf2*^-/-^ cells showed higher levels of PKCδ phosphorylation in all the conditions at both 4 and 8 hrs (Figs 7A and S7A). This increase in phosphorylation of PKCδ in *Nrf2*^-/-^ cells was not surprising since it is known that, in an oxidative milieu, such as NRF2 deficient cells, cysteine in the catalytic site of protein-tyrosine phosphatases (PTP) can be oxidized thus diminishing their activity [71]. Consequently, PTP substrates maintain a higher phosphorylated status [71,72]. Concomitantly, PP2 pretreatment resulted in notable reduction of NRF2 nuclear translocation and *Nqo1* gene expression in cells infected with both *Lgy*LRV1+ and *Lgy*LRV1- parasites (Fig 7B and 7C). Parasite entry was also affected by PP2 pretreatment, since lower infection rate was obtained with both *Lgy*LRV1+ and *Lgy*LRV1- parasites in the presence of PP2 (S7B Fig) suggesting the role of SFK in parasite phagocytosis. We validated PP2/SFK results in NRF2 activation by using PP3, a PP2 negative control, in *Lgy* infection. WT, *Nox2*^-/-^ and *Nrf2*^-/-^ BMDMs were pretreated with PP3 and subsequently infected with either *Lgy*LRV1+ or *Lgy*LRV1- parasites, or treated with tBHQ for 4 hrs. Similar levels of NRF2, PKCδ and SRC phosphorylation were detected in both *Lgy*LRV1+ and *Lgy*LRV1- infection in pretreated cells with PP3 (S7C Fig) confirming the specific inhibition of SFK by PP2. Altogether, we concluded that SFK and PKCδ were involved in NRF2 phosphorylation, translocation and activation in *Lgy* infection.

To have some additional information on which SFK could be implicated in the NRF2 activation pathway, we tested KB-SRC4, a c-SRC specific inhibitor and compared it to PP2 [73]. As shown in Fig 7D, we did not observe any effect of KB-SRC4 on the SFK/ PKCδ /NRF2 pathway in *Lgy*LRV1+ and *Lgy*LRV1- infected WT macrophages. Contrary to PP2, it had no effect on NRF2 protein and *Nrf2* gene expression, no significant effect on the downstream gene, *Nqo1*, on the number of parasites/cell, or on TNF-α secretion (Figs 7E, 7F, 7G and S7D). The only impact we could detect was on IL-6 secretion (S7E Fig).

Finally, we investigated whether activation of NRF2 in *Lgy*LRV1+ and *Lgy*LRV1- infected cells could be driven by a PERK/PKR/PI3K pathway culminating in AKT1 phosphorylation as reported in *L*. *amazonensis* infection [41]. Using MK2206, a specific AKT1 inhibitor, we did not observe any effect on NRF2 expression nor on the number of parasites/cell (Fig 7H–7I). To further verify that this activation pathway was not used in *Lgy* infection we also analyzed the phosphorylated status of eiF2α. In our experimental conditions, e.g., BMDMs differentiated into macrophages after M-CSF treatment, eiF2α was already phosphorylated in non-treated cells. No further modulation of its expression or of its phosphorylation status could be observed (S7F Fig). Thus, our data pointed to a defined pathway responsible for NRF2 activation including SFK and PKCδ.

## Discussion

In this study, we determined that activation of the NRF2 pathway by *L*. *spp*. occurred at a very early stage of infection independently of an endosymbiotic virus present in some *Leishmania spp*.. Driven by ROS, it implicated a functional NOX2 and a signaling pathway relying on SFK and PKCδ promoting NRF2 nuclear translocation and transcription of NRF2 target genes. We validated NOX2/SFK/PKCδ/NRF2 findings by recovering the *Nox2*^-/-^ phenotype with H_2_O_2_. We also blocked NRF2 pathway activation by PP2, a pan-specific SFK inhibitor, however, with higher selectivity for Fyn, Hck and Lck than for other members of the SRC family. These data provided additional support to a recently described signaling pathway in which NRF2 activation is modulated by SRC/PKCδ following H_2_O_2_ stimulation [29]. Thus, activation of host cell NRF2 occurred upon infection with every *L*. *spp*. tested, was critical to control ROS and did not depend on LRV1, nor on TLR signaling.

The SFKs comprise several members (c-SRC, Lck, Hck, Fyn, Yes, Fgr, Blk, Lyn and Frk) of which Hck, Fgr, and Lyn are preferentially expressed in myeloid cells. They can harbor different sensitivity to selective inhibitors. In our study, we not only used PP2 but also KB-SRC4, reported as a highly selective c-SRC kinase inhibitor [73]. It has been developed to only efficiently block c-SRC, with a >40-fold higher selectivity for c-SRC than for Lyn, Hck and Fyn [73]. In a side-by-side comparison using a pan-anti-SRC (Y416) antibody, we showed that KB-SRC4 had no effect on NRF2 activation whereas PP2 affected phosphorylation of SRC family members at Y416, blocked PKCδ phosphorylation, NRF2 nuclear translocation and transcription of downstream genes. Since KB-SRC4 is described to specifically block c-SRC our data suggested that the SFK member participating in NRF2 activation was not c-SRC but likely another member of the family such as Lyn, Hck or Fyn. Of course, we cannot exclude that, with the exception of c-SRC, several SFKs participate directly or indirectly to NRF2 activation.

Further characterization of the function of specific SFK(s) in *Leishmania* infections is important not only for their role in NRF2 activation but also in inflammation. In *Lgy*LRV1+ infected cells, indeed, PP2 and KB-SRC4 blocked secretion of TNF-α and IL-6, respectively. Although TNF-α and IL-6 are both produced in the hyper-inflammatory condition of *Lgy*LRV1+ infection, their regulation is different as PP2 can block secretion of TNF-α without affecting IL-6 levels [74,75]. Thus, if we consider different selectivity of KB-SRC4 and of PP2, secretion of the two cytokines could be regulated by different SFKs. Additional investigation is necessary to define the role of each of the SFKs and whether and how they could be specifically blocked. As possible therapeutic approach, it could be useful to block IL-6, which is essential in the initiation of the dissemination of the disease in LRV infection [10] without altering TNF-α and its anti-*Leishmania* effect.

In *Lam* infection, NRF2 activation depends on a PKR and PI3K/AKT pathway [40] and can also be induced by phosphorylation of PERK implicating increased phosphorylation of eiF2α and culminating in AKT1 phosphorylation important for NRF2 activation and increased expression of NRF2-downstream genes such as *Hmox1* [41] which could play a role in parasite survival. In our report, we provided evidence that, using conditional knocked-out mice, HO-1 was not implicated in a differential survival in infected mice with *Lgy*LRV1+ or *Lgy*LRV1- parasites.

Our data showed that, *in L*. *guyanensis* infection, AKT1 was not essential for NRF2 expression. Furthermore, we observed that eiF2α was already phosphorylated even in non-infected cells likely by the treatment with M-CSF used to derive bone marrow macrophages. These results suggested that in our experimental *Lgy* infection model, the PERK/PKR/AKT1 axis was not important. We should point out, however, that both pathways are not mutually exclusive and possibly depend on particular features of the infecting species and the initial activation step. In *Lam* a role for TLR-2 in PERK-dependent NRF2 [41] activation is detailed whereas we could exclude such a role in NRF2 activation by infecting *MyD88*^-/-^ mice with *Lgy*. These results provided some evidence for a different activation mode of NRF2 depending on the infecting species at least between *Lam* and other *L*. *spp*.. Such differences are already known in terms of ROS production, detoxification and sensitivity which differ between *L*. *spp*. and in infected host cells. For example, there is more HO-1 expressed in macrophages of CBA mice treated with the abundant cell surface glycolipid LPG of *Lam* than with LPG of *Lmj*. Iron metabolism is different in CBA macrophages infected with *Lam* or *Lmj*, possibly correlating to differential susceptibility to infection in CBA mice [45].

*Leishmania* parasite-triggered phagocytosis is described to promote the assembly of the NOX2 complex [76] and NOX2 induction of NRF2 pathway was likely independent from phagocytosis since pharmacological impairing of phagocytosis by cytochalasin D did not have an effect on the NRF2 pathway in *Lgy* infection. Interestingly, phagocytosis was significantly altered by PP2 treatment but not by KB-SCR4. Previous studies have highlighted the role of SFKs, Hck, Fgr and Lyn in phagocytosis of *Lam* amastigotes via FcγR [18]. Which SFK is implicated in promastigotes phagocytosis should be further evaluated in infection with *Lgy* and its endosymbiont. This could be relevant to understand a possible link between co-infections, phagocytosis, NRF2 and hyperinflammation.

When macrophages were exposed to PFA-treated parasites, NRF2 and its downstream genes, *Hmox1* and *Nqo1*, were not, or only weakly activated, whereas activation occurred with heat- or UV-treated parasites. These data supported a role for proteinous components of the parasite cell surface as initiators of the NRF2 response. None of these treated parasites entered macrophages as measured by high-content imaging. These findings suggested that the initial contact with live parasites was sufficient for activating NRF2 pathway in macrophages, irrespectively of the species and of the presence of LRV1, that phagocytosis was not absolutely required for NRF2 activation and that NRF2 could also be activated by a TLR-2 agonist such as zymosan. We cannot exclude however that NRF2 activation could be solely due to a simple contact and ROS production activating SRC kinase [29] but can exclude an essential role for phagocytosis in NRF2 activation.

Concomitantly, we observed that brief exposure to live or metabolically active parasites such as UV-treated parasites was sufficient to increase mitochondrial respiration in macrophages, which was attributed to the NRF2 pathway and phagocytosis. Immune cells reprogram their metabolism in order to protect against infection by pathogenic microorganisms and it has been reported that NRF2 is involved in mitochondrial respiration [63,77]. The nature of the contact between the parasite and the host cell surface molecules requires further investigation but could be relevant to understand the rapid reprogramming of the metabolism of a cell facing an invading pathogen.

NRF2 pathway activation was similar for *Lgy*LRV1+ and *Lgy*LRV1- parasites in macrophages. However, we showed that the entry of *Lgy*LRV1+ into the host cell was crucial for inducing inflammation as cytochalasin D prevented the pro-inflammatory response induced by the dsRNA of *Lgy*LRV1+ acting on TLR-3 as previously reported [8]. Thus, contrary to *Lgy*LRV1-, which only activated NRF2, *Lgy*LRV1+ induced NRF2 and TLR-3-dependent inflammatory cytokines. This hyperinflammation was under the regulation of the p65 NF-κB subunit when infected with *Leishmania* parasites bearing LRV1, whereby host cells upregulated nuclear translocation of the p65 NF-κB subunit and TNF-α secretion. Thus, in our study, absence of NRF2 had an impact only when NF-κB was already activated via TLR3 as we could not detect significant level of TNF-α or IL-6 in *Nrf2*^-/-^ macrophages infected with *Lgy*LRV1-. This indicated that in most infections with *Leishmania* parasites, in the absence of any viral co-infection, ROS are controlled by NRF2 and are likely not sufficient to activate NF-κB as shown in other model systems [50].

TNF-α cytokine has a crucial role in control of lesion development since IFN-γ is likely not sufficient for an effective protective function [58]. This explains why *Nrf2*^-/-^ mice had significantly more TNF-α and therefore reduced footpad swelling, and parasite burden possibly associated with increased sensitivity to TNF-α in *Nrf2*^-/-^ mice [78]. Similarly, dKO mice had increased cellular infiltration in the footpads and showed augmented cartilage destruction in the tail. Studies with *Nrf2*^-/-^ mice in the context of spinal cord injury displayed higher levels of TNF-α and MMP9 activity [79]. These data correlated to TNF-α quantities and tissue destruction observed in mucosal lesions of MCL patients and thus represent a clear risk factor for disease development [12]. The data also showed that it was in the interest of the host and of the parasite to control TNF-α levels and to avoid uncontrolled inflammation. Whether, and how the TNF-α detrimental effect in lesions relates to the production of perforin by cytotoxic CD8+ T cells in infected patients [80] remains to be determined.

**Fig 8:**
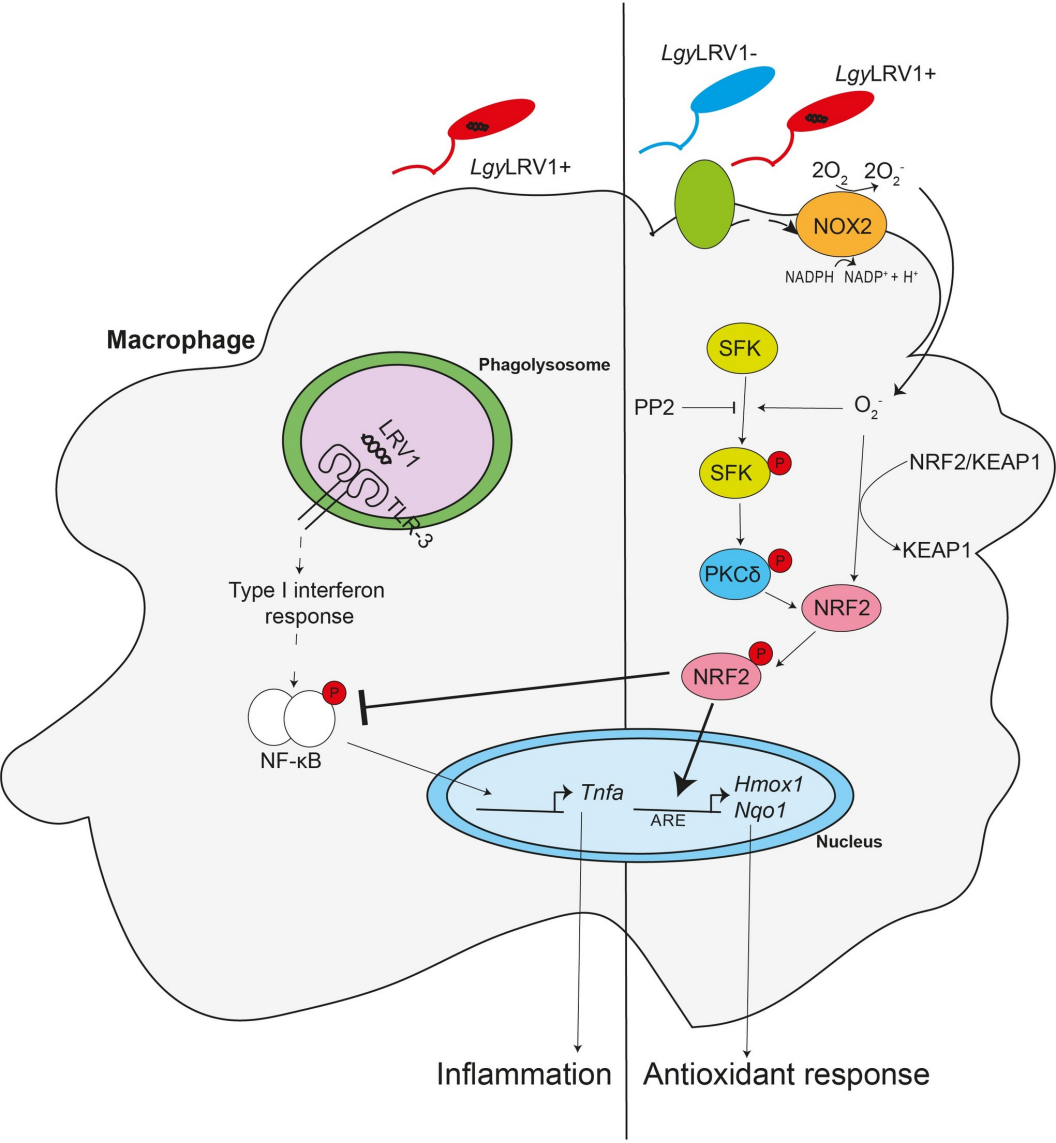
NRF2 protects from oxidative stress and hyperinflammation in *Lgy* infection. During infection with *Lgy* parasites, the NRF2 pathway is activated independently of LRV1 by the production of oxidative species by NOX2. NOX2 activation triggers PKCδ phosphorylation by SFK resulting in the release of NRF2 from its negative regulator KEAP1. Stabilized NRF2 translocates to the nucleus favoring the antioxidant response. Concomitantly, stabilized NRF2 blocks the induced pro-inflammatory response activated by LRV1. Thus, NRF2 activation results in activation of the antioxidant response and restriction of the pro-inflammatory response in *Lgy* infection.

In conclusion, even if there are still some questions on the precise role of the different members of SFKs in myeloid cells, on the cell surface molecule(s) participating in a rapid activation of NRF2 and on metabolic adaptation in the infected cell, our study described the molecular mechanisms underlying NRF2 activation in *L*. *spp*. (Fig 8*)*. ROS activation of NRF2 could occur either by phagocytosis of parasites and NOX2 or by simple contact of parasites with the host cell surface. Both initial signals culminate in phosphorylation of SFK, PKCδ and NRF2, which has been released from KEAP1 which is inactivated by oxidative modifications of specific residues. The importance of kinases in this activation process could offer potential therapeutic targets to treat and prevent leishmaniasis. In this respect, inhibition of the SRC kinase family was shown to decrease lesion size [18]. We also showed that parasite contact and the oxidative burst are sufficient to boost the antioxidant and anti-inflammatory response enhanced by the NRF2 pathway in macrophages infected with *Leishmania* parasites. However, blocking or altering the NRF2 pathway could unleash inflammation, favor dissemination of the infection and cause tissue destruction as observed in MCL patients. Thus, these results revealed also that *Lgy* parasites not only take advantage of the antioxidant response from the host NRF2 activation but also from the host controlled inflammatory response to avoid excessive production of TNF-α.

## Materials and methods

### Experimental models

#### Ethics statement

All animal protocols described in this report were approved by the Swiss Federal Veterinary Office (SFVO), under the authorization number VD 2113. Animal handling and experimental procedures were undertaken with strict adherence to the ethical guidelines set out by the SFVO and under inspection by the Department of Security and Environment of the State of Vaud, Switzerland.

#### Mice

WT (C57BL/6) mice were purchased from Envigo (Netherlands). *Tlr3*^-/-^ mice [81] were acquired from Prof. S. Akira (Osaka University, Japan) via P. Launois (WHO-IRTC, Lausanne, Switzerland). *Nrf2*^-/-^ [23], *Hmox1*fl/flxLysm^+/-^ and *Hmox1*fl/flxLysm^-/-^ mice [57] were obtained from Prof. M. Yamamoto (Tohoku University Graduate School of Medicine, Japan). *Ifng*^-/-^ mice [82], *Nox2*^-/-^ mice [83] and *Inos*^-/-^ [84] mice were purchased from the Jackson Laboratories. *Ifng*x*Nrf2* dKO mice were produced by intercrossing *Ifng*^-/-^ and *Nrf2*^-/-^ mice for 3 generations. Mice were genotyped by PCR using tissue-isolated genomic DNA and the KAPA Mouse Genotyping Kit (KAPA Biosystems). The oligonucleotides used for genotyping are specified in S1 Table. Mice were maintained under a pathogen-free environment at the animal facility of the Center of Immunity and Immunology, Lausanne (Switzerland). Food (SAFE or KILIBA NAGAF) and water (Local water filtered and autoclaved after acidification or Innovive Aquavive) were provided ad libitum. Light cycle was maintained with 13 hrs light and 11 hrs darkness, temperature was set at 21°C ± 2 and humidity was kept at 55% ± 10. Mouse experiments were performed in a P2 pathogen animal facility at the Center of Immunity and Immunology, Lausanne (Switzerland). The mice and all experiments were conducted under the guidelines set by the State Ethical Committee for the utilization of laboratory animals.

#### Parasites

Two isogenic clones of *L*. *guyanensis* (*Lgy*) containing or lacking LRV1 (*Lgy*LRV1+ (LRV1^+^ *Lgy*M4147/SSU:IR2SAT-LUC(b)c3) and *Lgy*LRV1- (LRV1^-^ *Lgy*M4147/SSU:IR2SAT-LUC(b)c3), respectively) were used. Both clones were described previously [56] and derived from the LRV1+ parent strain, *Lgy* M4147 (MHOM/BR/75/M4147). These parasites express similar levels of a firefly luciferase (ffLUC) gene (5x10^7^ photons/sec/10^6^ parasites) integrated into the ribosomal RNA locus small subunit (SSU). Here we generated isogenic *Lgy*M4147 LRV1+ parasites expressing both ff/LUC and mCherry as follows. The mCherry ORF was amplified by PCR using SMB7961 and SMB7962 primers (S1 Table) from PCR-Blunt-mCherry template (B7710) and after digestion with BgIII, inserted into the BgIII site of pIR4HYG-LUC(A) (strain B7714) or pIR4BSD-LUC(A) (strain B7715), yielding pIR4HYG-LUC(A)-mCherry(b) or pIR4BSD-LUC(A)-mCHerry(b) (strains B7716 or B7717, respectively). Following digestion with SwaI, DNAs were transfected into the *Lgy* strains above [56] by electroporation as previously described [85], and clonal lines obtained by plating on semisolid M199 medium containing hygromycin B (200 *μ*g/ml) or blasticidin (10 μg/ml). After expansion, parasites were grown one additional passage in the absence of noreothricin and in the presence of 100 μg/ml hygromycin B or 5 μg/ml blasticidin. This protocol was designed to simultaneously introduce the LUC-mCherry construct (HYG marker) and drop out the previous LUC construct (SAT marker). Many colonies were obtained, screened for the presence of *HYG* or *BSD* and loss of *SAT* markers, and then for both mCherry and LUC expression. Overall, 8/43 mCherry+ clones tested lost the *SAT* marker, suggesting a relatively frequent marker exchange by this protocol. Several clonal lines for each marker/virus were then passaged once through mice prior to use thereafter. In these studies, clonal line 8 was used for LgyM4147/SSU:IR4BSD-LUC(a)-mCherry(b)/LRV1+.

Three strains of *L*. *infantum* (*Linf*) were used. The first one was isolated from a dog in Spain (MCAN/ES/98/LLM-722) [86], whereas the second one was recovered from a human patient from Switzerland (MHOM/CH/2016/BELA). The strains will be referred as *Linf* S1 and *Linf* S2 respectively, as reported previously [87]. The third *Linf* strain, referred as *Linf* S3, corresponds to MHOM/MA67/IT/MAP263 strain [88]. Other strains were used in this study such as *L*. *donovani* (*Ldo*) (MHOM/SD/62/1S-Cl2D) [89], *L*. *aethiopica* (*Lae)* (MHOM/ET/2008/LDS372) [90], *L*. *braziliensis* (*Lbraz*) (MHOM/BR/00/LTB325) [91], *L*. *amazonensis* (*Lam*) (LTB0016) [60], *L*. *panamensis* (*Lpa*) (MHOM/CO/86/1166) [91], *L*. *tropica* (*Ltr*) (MHOM/SY/91/LEM2379) and *L*. *mexicana* (*Lmex*) (MYNC/BZ/62/M379) [92]. *L*. *major* (*Lmj)* (MRHO/IR/75/ER (IR75)) [93] strain was also used. *Lgy*, *Lbraz*, *Lam* and *Lae* parasites were cultured in Schneider’s Drosophila medium (PAN BIOTECH) supplemented with 20% heat inactivated Fetal Bovine Serum (FBS, Gibco), 1% HEPES Buffer, 1% Penicillin-Streptomycin (P/S) solution (BioConcept), 0.6 μg/ml of 6-Biopterin (Sigma-Aldrich) and 0.1% Hemin-Folate solution (Sigma-Aldrich, Fluka). *Lmj*, *Linf* S2, *Ldo*, *Lmex* and *Ltr* parasites were cultured in Medium 199 (M199, Gibco) supplemented with 20% heat inactivated FBS, 1% HEPES Buffer, 1% P/S solution, 0.6 μg/ml of 6-Biopterin and 0.1% Hemin-Folate solution. *Linf* S1, *Linf* S3 and *Lpa* were cultured in RPMI 1640 Medium (Gibco) complemented with 10% heat inactivated Fetal Bovine Serum (FBS) and 1% P/S solution. In general, the parasites were maintained in culture *in vitro* as promastigotes at 26°C and 5% CO_2_ for a maximum of 5 passages. Each passage yielded stationary- phase promastigotes after 6 days. For some experiments, *Lgy* parasites were killed by 4% paraformaldehyde (PFA) (Fluka) fixation for 15 min and washed 2x with DPBS1x. *Lgy*LRV1+ parasites were also killed with either UV treatment for 1 hr or 95°C heat treatment for 10 min and washed 2x with DPBS1x. All reagents used for parasite culture are detailed in S2 Table.

### Method details

#### Macrophage infection

Bone marrow cells were extracted from the femurs and tibias of naïve mice. The extracted cells were differentiated into BMDMs for 6 days at 37°C and 5% CO_2_ in 10 ml of Dulbecco’s modified Eagle’s medium (DMEM, Gibco) supplemented with 10% FBS, 1% HEPES, 1% P/S and 50 ng/ml Recombinant Mouse Macrophage Colony Stimulating Factor (rm M-CSF, ImmunoTools). 3 days after, 5 ml of complete DMEM with rm M-CSF was added to the culture. Differentiated BMDMs were seeded on culture plates at a concentration of 1.25 x 10^6^ cells/ml and incubated overnight. BMDMs were infected with stationary-phase promastigotes at multiplicity of infection (MOI) of 10 parasites per macrophage for 15 min, 1, 2, 4, 8 or 24 hrs at 35°C. Alternatively, BMDMs were kept at 37°C when infected with *Lmj*, *Linf*, *Ldo*, *Lmex* and *Ltr* parasites. BMDMs were also treated with *tert*-Butylhydroquinone (tBHQ) at 10 μM (Sigma-Aldrich), poly I:C at 2 μg/ml, zymosan at 1 μg/ml, β-glucan peptide (BGP) at 100 μg/ml (Invivogen), or H_2_O_2_ (Sigma-Aldrich) at 250 μM, 100 μM and 50 μM at 35°C. In addition, in some cases BMDMs were pretreated prior to *Leishmania* infection for 1 hr at 37°C with Cytochalasin D at 40 μM, PP2 at 100 μM (Sigma-Aldrich), PP3 at 10 μM (Tocris), KB-SRC4 at 100 μM (R&D Systems), MK-2206 at 5 μM (Apexbio) or DMSO (AppliChem GmbH) as control. All reagents used for macrophage culture are detailed in S2 Table.

#### Cytokine analysis by ELISA

The concentrations of TNF-α and IL-6 (Invitrogen) in collected supernatants from 24 hr-treated BMDMs were determined using enzyme-linked immunosorbent assay (ELISA) in duplicate following the manufacturer’s instructions. The plates were read on a Synergy HT Multi-Mode Reader (BioTek). Wavelength correction and background signal were subtracted from the absorbance values.

#### Mouse infection and *in vivo* bioluminescence imaging

Age-matched (6-8-week-old) female mice were infected subcutaneously in both hind footpads with 1 x 10^6^ stationary-phase *Lgy*LRV1+ or *Lgy*LRV1- parasites in 50 μl of Dulbecco’s Phosphate-Buffered Saline (DPBS, Gibco). Change in footpad thickness was measured weekly using a Vernier caliper as proxy for disease progression. Immunocompromised mice, such as *Ifng*^-/-^ and dKO mice, received constant pain medication (1g/L Dafalgan (Upsa) diluted in drinking water) during the second half of their infection. *In vivo* parasite burden was quantified by intra-peritoneally injecting VivoGlo Luciferin (Promega) at a concentration of 150 mg/kg and measuring the bioluminescence produced in the mouse footpads with *In-Vivo* Xtreme II (BRUKER) as previously described [94]. Alternatively, *in vivo* ROS levels were measured by intra-peritoneally injecting Luminol sodium salt (Carbosynth) at a concentration of 200 mg/kg and following the same procedure as parasite burden quantification. The acquired images were analyzed using Molecular Imaging (MI) software (BRUKER). Regions of Interest (ROI) were set on the footpads and the bioluminescent signals were expressed in units of photons per second (P/s) [94].

#### Oxygen Consumption Rate (OCR) measurement

BMDMs were plated in Seahorse XF96 cell culture microplates (Agilent). After overnight incubation, cells were either infected with live, or exposed to heat-killed, UV-treated or PFA-fixed *Lgy*LRV1+ parasites, or infected with *Lgy*LRV1- or *Linf* S1 or *Linf* S3 parasites, or non-infected (Ø) or treated with zymosan particles (1 *μ*g/ml) or β-glucan peptide (BGP) (100 *μ*g/ml) for 15 min at 35°C and 5% CO_2_. To determine the cell’s metabolic activation after parasite infection, the oxygen consumption rate (OCR) was measured. Briefly, the cells were pre-incubated in 180 μl of assay medium (Seahorse XF DMEM pH 7.4 (Agilent) supplemented with 2 mM L-glutamine, 1 mM pyruvate and 25 mM glucose (Gibco)) for 1 hr at 37°C in an CO_2_ -free incubator. OCR rates over time were measured using Seahorse XFe96 Analyzer (Agilent). Seahorse Cell Mito Stress Test protocol (User guide Kit 103015–100, Agilent) was employed. Results were analyzed using Wave Desktop software (Agilent). Measurements were normalized to total protein concentration per well. Cells were lysed with a mixture of 5x RIPA Buffer IV (Bio Basic) and a complete protease inhibitor cocktail tablet (Roche) in H_2_0. Protein concentration was quantified using Pierce BCA Protein Assay Kit (Thermofisher Scientific) following manufacturer’s instructions. All reagents used for OCR measurement are detailed in S2 Table.

#### Western blot analysis

Cells were lysed in 100 μl of 1.5x Laemmli’s Sample Buffer in H_2_O after 1, 2, 4, 8, 24 hrs post-treatment and incubated at 95°C for 3 min. Cell lysates were size-fractioned by 8% SDS-PAGE gels and wet-transferred to nitrocellulose membranes. Membranes were blocked with 5% non-fat dry milk in Tris buffered saline with 0.1% Tween-20 (TBST) at room temperature. Immunoblotting was conducted overnight at 4°C with 1:5000 anti-β-Galactosidase (Promega), 1:1000 anti-NRF2 (Abcam), 1:10,000 anti-γ-TUBULIN (Sigma-Aldrich), 1:1000 anti-phospho NRF2 (BIOMATIK), 1:1000 anti-SRC, 1:1000 anti-phospho SRC, 1:1000 anti-PKCδ, 1:1000 anti-phospho PKCδ, 1:1000 anti-AKT, 1:1000 anti-phospho AKT, 1:1000 anti-eiF2α and 1:1000 anti-phospho eiF2α (Cell Signaling) primary antibodies and by incubating the membranes with 1:2000 goat anti-rabbit IgG (H+L) HRP or 1:2000 goat anti-mouse IgG (H+L) HRP (Promega) secondary antibodies for 1 hr at room temperature. The membranes were washed with TBST in between antibody incubation. The immunoblots were revealed by ECL Western Blotting detection reagent (GE Healthcare Life Sciences). Alternatively, ECL Select Western Blotting detection reagent (GE Healthcare Life Sciences) was used for revealing NRF2 and phosho-PKCδ proteins. Immunoblots were visualized with Fusion Solo S (Vilber) or by films (Amersham Hyperfilm, GE Healthcare Life Sciences) which were developed using a radiograph (SRX-101a, Konika Minolta). Relative protein levels were determined in some cases by band quantification using the Gel analysis method in Image J software. All antibodies used are detailed in S3 Table.

#### RNA isolation and RT-qPCR

Footpads or tail pieces from WT, *Nrf2*^-/-^, *Ifng*^-/-^ and dKO mice were recovered at the end of *Lgy*LRV1+ infection and snap-frozen on dry ice. For RNA isolation, the samples were submerged in TRI Reagent (Molecular Research Center, inc) and stainless beads (Qiagen) were added to favorize tissue disruption using TissueLyser system (Qiagen). RNA was isolated by chloroform/isopropanol/ethanol phase separation as described previously [95]. RNA quantity and quality were analyzed using NanoDrop 2000 (ThermoFisher Scientific). Alternatively, BMDMs were lysed with PRImeZOL Reagent (Canvax) after either 1, 2, 4, or 8 hrs post-treatment. RNA from cells was extracted using Direct-zol-96 RNA (Zymo Research) following the manufacturer’s description. cDNA was generated using SuperScript II Reverse Transcriptase (Invitrogen). Real-time quantitative PCR (RT-qPCR) was performed using LightCycler 480 SYBR Green I Master (Roche), with 0.5 μM primer pairs, on LightCycler 480 (Roche). Primers used and sequences are listed in S1 Table. Gene expression was analyzed using the threshold cycle (C_T_) method 2^-ΔΔCt^. Data were normalized to *L32* expression and samples were calibrated to the expression of the gene of interest in medium-treated in BMDMs, whereas the fold induction expression in the footpads or tail pieces was normalized to either WT mice, for WT and *Nrf2*^-/-^ comparisons, or *Ifng*^-/-^ mice, for *Ifng*^-/-^ and dKO comparisons. All reagents used for RNA isolation and RT-qPCR are detailed in S2 Table.

#### Confocal microscopy

BMDMs were seeded in 15μ-slide 8 well plates (IBIDI). After overnight incubation, cells were infected with *Lgy* parasites or treated with 10 μM tBHQ (Sigma-Aldrich) for 8 hrs. Cells were fixed with PFA 4% for P65 protein or, 4% PFA and 100% methanol consecutively for NRF2 protein. Next, cells were blocked and permeabilized in PBS solution with 5% donkey serum (BIO-RAD), 0.5% Bovine Serum Albumin (BSA, Sigma-Aldrich) and 0.3% Triton X-100 (Roche), for 1 hr at room temperature. Primary antibodies: 1:200 anti-NRF2 (Abcam), 1:200 anti-LAMIN A/C (Abcam) or 1:400 anti-P65 (Cell Signaling), were incubated overnight at 4°C. Donkey Secondary antibodies conjugated to Alexa 488 and 554 (Invitrogen) and DAPI (Molecular Probes) were incubated for 1 hr at room temperature. A PBS solution with 0.1% Triton X-100 was used as washing buffer. For acquisition, six to eight representative images were acquired per well on LSM 880 confocal microscope (Zeiss), using Plan Neofluar 63x oil objective. Images were obtained using Zen Black (Zeiss) software. NRF2 and P65 nuclear translocation were quantified with IMARIS software (Oxford Instruments). Both NRF2 and P65 cytoplasmic emission and nuclear signal were measured by cells with an organelle module. LAMIN A/C was used as cell nuclear marker instead of DAPI for quantification, NRF2 and P65 nuclear translocation are expressed as the ratio of nuclear NRF2 or P65 over cytoplasm. Additionally, parasite cellular internalization was determined with live cell imaging at 8 hrs post-infection with mCh*Lgy*LRV1+ parasites combined with Differential Interface Contrast (DIC) microscopy on LSM800 confocal microscope (Zeiss), using Plan Apochromat 20x objective. Images were obtained using Zen Blue (Zeiss) software. All reagents used for confocal microscopy and antibodies are detailed in S2 and S3 Tables, respectively.

#### High-content microscopy

BMDMs were plated in μ-Plate 96 Well Black (IBIDI). After overnight incubation, cells were infected with *Lgy* parasites for 8 or 24 hrs. Cells were then fixed with 4% PFA, stained with DAPI (Molecular Probes) to visualize the nuclei of parasites and macrophages and washed with PBS using EL406 plate washer (Biotek). Twenty-five images (5x5 square) from each well with a 40x lens were acquired using ImageXpress Micro XL (Molecular Devices). Cell and parasite numbers were segmented and counted using MetaXpress custom Module Editor (Molecular Devices) as described previously [96]. Data is expressed as parasite/cell as a proxy of parasite burden. Alternatively, automated microscopy was also used to determine NRF2 nuclear translocation. Cells were pretreated with 100 μM PP2 or 40 μM Cytochalasin D for 1 hr prior to *Lgy* infection for 8 hrs. NRF2 and DAPI staining were performed as described in *Confocal microscopy*. Twenty-five images (5x5 square) from each well with 40x Plan Apochromat lens were acquired using ImageXpress Micro Confocal (Molecular Devices). To quantify the NRF2 nuclear signal translocation the MetaXpress Custom module editor was used to segment the cell nucleus (using DAPI signal) and the cell body (using NRF2 signal), using the same method as to count the parasite nuclei. The average NRF2 signal intensity has been extracted from both area (Nuclei and Cytoplasm) and a ratio has been calculated and normalized to the medium control. All reagents used for high-content microscopy and antibodies are detailed in S2 and S3 Tables, respectively.

#### ROS production by time-lapse microscopy

CFSE Fluorescent Cell labelling (Abcam) was performed according to the manufacturer’s description before plating the cells in 15μ-slide 8 well plates (IBIDI) using DMEM without phenol red (Gibco). After overnight incubation, cells were infected with *Lgy* parasites and Dihydrorhodamine 123 (DHR, Invitrogen) was added simultaneously. Live cell imaging was performed on LSM800 confocal microscope (Zeiss) using Plan Apochromat 20x objective. Images were acquired with the pinhole totally open to scan in non-confocal mode in order to minimize the laser intensity on the cells. Six images per condition were acquired every 20 min during 14 hrs at 35°C and 5% CO_2_ using full enclosure PECON incubator using Zen Blue (Zeiss) software. ROS level accumulation in BMDMs were quantified using Image J (NIH) by encoding a macro in RGB batch mode. Threshold was set for CFSE staining, which was used to define the ROI for measuring the cellular DHR signal as a proxy of ROS production. Results are expressed as Average intensity showing the mean of all the positions per condition at each time-point. Additionally, cell count was determined with CFSE staining and it is expressed as the mean of all the positions per condition at each time-point. All reagents used for ROS production measurement are detailed in S2 Table.

#### Histological staining

Footpad lesions and 5 mm long tail pieces were fixed in 4% PFA. Tail pieces were also decalcified with Shandon TBD-1 Decalcifier (Thermofisher Scientific) for 24 hrs at 4°C. All samples were embedded into paraffin and 5 μm transversal sections were obtained. Sections were stained with Masson’s Trichrome Blue by the Mouse Pathology Facility at the University of Lausanne. Entire sample area was acquired on AxioScan.Z1 scanner (Zeiss) with Plan Apochromat 20x/0.80 objective using Brightfield contrast or NanoZoomer S60 (Hamamatsu Photonics K.K.) scanner with Nikon Plan Apochromat 20x/0.75 objective using Brightfield contrast. Images were obtained using the following software: Zen Blue (Zeiss) for AxioScan.Z1 scanner or NPD.scan3.3 (Hamamatsu Photonics K.K.) for NanoZoomer S60 scanner. Representative images of the sections are shown.

#### Statistical analysis

All graphs generated and related statistical analysis were performed using GraphPad Prism. Unpaired Student’s test was used for single-point analysis on bar graphs; while repeated-measure Two-way ANOVA test was used for x/y curves, with Bonferroni’s post-test correction. Significance was reached with p values ≤ 0.05. p values are shown as * for p < 0.05, ** for p < 0.01, *** for p < 0.001 and **** for p < 0.0001. NS, non-significant statistical difference.

The numerical data used in all figures are included in S1 Data.

## Supporting information

S1 Fig Related to Fig 1. ***Lgy* parasites are sufficient to upregulate the NRF2 pathway without LRV1 and *L*. *spp*. infection in NRF2 expression**. A and B) WT and *Nrf2* ^-/-^ BMDMs were infected with *Lmj*, *Ltr*, *Lae*, *Lmex*, *Lam*, *Lgy*LRV1+, *Lgy*LRV1-, *Lbr*, *Lpa*, *Linf* S1, *Linf* S2 or *Ldo* parasites. Negative and positive controls for NRF2 activation of non-treated (Ø), or tBHQ-treated (10 μM) were performed concurrently. NRF2 protein levels were assessed by Western Blot at 8 hrs using anti-NRF2 and anti-γTUBULIN (A) and *Nqo1* relative RNA levels were normalized to *L32* housekeeping gene assessed by RT-qPCR using the 2^ΔΔCT^ method (B). C) WT cells were infected with *Lmj*, *Ltr*, *Lae*, *Lmex*, *Lam*, *Lgy*LRV1+, *Lgy*LRV1-, *Lbr*, *Lpa*, *Linf* S1, *Linf* S2 or *Ldo* parasites for 8 hrs. DNA (blue) was stained with DAPI for assessing parasite internalization at 63x using confocal microscopy. Scale bar represents 10 μm. D) *Nrf2* ^-/-^ BMDMs were infected with *Lgy*LRV1+, *Lgy*LRV1-parasites. Negative and positive controls for NRF2 activation of non-treated (Ø), or tBHQ-treated (10 μM) were performed concurrently. β-galactosidase (β-GAL) protein levels were assessed by Western Blot using anti- β-GAL and anti- γTUBULIN antibodies at the indicated time-points. E) WT cells were infected with either *Lgy*LRV1+ or *Lgy*LRV1- or non-treated (Ø), or tBHQ-treated (10 μM) for 1, 2, 4 and 8 hr time-points. *Nrf2* relative RNA levels were normalized to the *L32* housekeeping gene assessed by RT-qPCR using the 2^-ΔΔCT^ method. F) WT and *Nrf2* ^-/-^ cells were infected with either *Lgy*LRV1+ or *Lgy*LRV1- or non-treated (Ø), or tBHQ-treated (10 μM) for 1, 2, 4 and 8 hr time-points. *Hmox1* and *Nqo1* relative RNA levels were normalized to *L32* housekeeping gene assessed by RT-qPCR using the 2^-ΔΔCT^ method. G and I) WT, *Tlr3* ^-/-^ and *Nrf2* ^-/-^ BMDMs were infected with either *Lgy*LRV1+ or *Lgy*LRV1- parasites. Negative and positive controls of non-treated (Ø), tBHQ (10 μM), poly I:C (I:C, 2 μg/ml), TLR3 agonist, were performed concomitantly. NRF2 protein levels were assessed by Western Blot at 4 hrs using anti-NRF2 and anti-γTUBULIN antibodies (G) and *Hmox1* and *Nqo1* relative RNA levels were normalized to the *L32* housekeeping gene assessed by RT-qPCR using the 2^-ΔΔCT^ method at 8 hrs post-infection (I). H) WT, *MyD88* ^-/-^, *Nrf2* ^-/-^ BMDMs were infected with *Lgy*LRV1+, *Lgy*LRV1-parasites or non-treated (Ø), or tBHQ-treated (10 μM). Cell lysates after 4 hrs post-infection were analyzed by Western Blot using anti-NRF2 and anti-γTUBULIN. J). Cell count related to Fig 1D) using CFSE-labelling of cells during ROS quantification with DHR 123 by time-lapse microscopy during 14 hrs. Cells were quantified using CFSE staining by Image J software. Representative blots and images from three (A, D and G), two (H) and one (C) independent experiments are shown. Data represented as mean ± SEM from a pool of three independent experiments (B, E, F and I), or one of three independent experiments (J). Significance was calculated by Student’s t test. Not significant (ns), * p < 0.05 and ** p < 0.001.
(TIF)

S2 Fig Related to Fig 2. **NRF2 controls inflammation in *Lgy*LRV1+ infection by blocking proinflammatory cytokine *Tnfa* transcripts**. WT, *Nrf2*^-/-^, *Ifng*^-/-^, *Ifng*x*Nrf2* dKO (dKO), *Hmox1*fl/flxLysm^+/-^ or *Hmox1*fl/flxLysm^-/-^ mice were infected in both hind footpads with 1x10^6^ of *Lgy*LRV1+ parasites. A and B) Relative RNA expression levels of NRF2 target genes *Hmox1* (A) and *Nqo1* (B) in the footpads of WT and *Nrf2*^-/-^ at 3 weeks post-infection using RT-qPCR normalized to *L32* housekeeping gene. C) Footpad swelling evolution was measured weekly as a proxy for disease progression for *Hmox1*fl/flxLysm^+/-^ or *Hmox1*fl/flxLysm^-/-^. D) Relative RNA expression levels of *Tnfa* transcripts in the footpads of WT and *Nrf2*^-/-^ at 3 weeks post-infection using RT-qPCR normalized to *L32* housekeeping gene. E-G) Relative RNA expression levels of *Hmox1* (E), *Nqo1* (F) and *Tnfa* transcripts (G) in the footpads of *Ifng*^-/-^ and dKO at 3 weeks post-infection using RT-qPCR normalized to *L32* housekeeping gene. H and I) Relative RNA expression levels of *Tnfa* transcripts in the footpads (H) or tail (I) of *Ifng*^-/-^ and dKO at 8 weeks post-infection using RT-qPCR normalized to *L32* housekeeping gene. J) Representative X-Ray images indicating tail tissue and bone destruction at week 8 for WT and *Nrf2*^-/-^ mice. K) Representative images of Masson’s trichrome staining of footpad sections at week 3 for WT, *Nrf2*^-/-^, *Ifng*^-/-^ and dKO mice at 20x on an automated slide scanning microscope. Collagen (blue), nuclei (black), muscle and cytoplasm (red) are represented. Scale bar represents 100 μm. Data show mean ± SEM from the pool of one (A-G and K) two (H and I) or three (J) independent experiments (n = 5–12 mice). Significance was calculated by Student’s t test. * p < 0.05 and *** p < 0.001.
(TIF)

S3 Fig Related to Fig 3. **NRF2 expression controls NF-κB -inflammation in *Lgy*LRV1+ infected macrophages**. Relative RNA expression levels of *Nfkb1* gene at 8 hrs in WT and *Nrf2* ^-/-^ cells infected with either *Lgy*LRV1+ or *Lgy*LRV1- parasites, non-treated (Ø), tBHQ-treated (10 μM), or poly I:C (I:C, 2 μg/ml), assessed by RT-qPCR and normalized to the *L32* housekeeping gene. Data expressed as mean ± SEM from two independent experiments. Unpaired Student’s t test was used to measure statistical significance. * p < 0.05.
(TIF)

S4 Fig Related to Fig 4. **NOX2 regulation of the NRF2 pathway is conserved along *Leishmania* parasites**. BMDMs from WT, *Nox2* ^-/-^, *Inos* ^-/-^ and *Nrf2* ^-/-^ mice were infected with either *Lmj*, *Ltr*, *Lae*, *Lmex*, *Lam*, *Lgy*LRV1+, *Lgy*LRV1-, *Lbr*, *Lpa*, *Linf* S1, *Linf* S2 or *Ldo* parasites. Negative and positive controls for NRF2 activation of non-treated (Ø), or tBHQ-treated (10 μM) were performed concurrently. A, C and D) Immunoblotting of NRF2 and γTUBULIN proteins in cell lysates at 4 (B and C) and 8 (A) hrs. Relative NRF2 levels were determined by band quantification using Image J software and given as NRF2 over γTUBULIN. B) Relative RNA expression levels of *Nrf2* were normalized to *L32* housekeeping gene assessed by RT-qPCR using the 2^-ΔΔCT^ method in WT and *Nox2* ^-/-^ cells. Representative blots and their quantification from three independent experiments are shown. Data expressed as mean ± SEM from two independent experiments. Unpaired Student’s t test was used to measure statistical significance. Not significant (ns) and * p < 0.05.
(TIF)

S5 Fig Related to Fig 5. **The NRF2 pathway is not abrogated by phagocytosis blockage in *Lgy* infection**. A and B) WT cells were pretreated with DMSO or Cytochalasin D (Cyt D, 40 μM) for 1 hr and infected with *Lgy*LRV1+ or *Lgy*LRV1- parasites or stimulated with tBHQ (10 μM) or medium-treated (Ø). A) Cells were lysed at the 8 hr time-point and immunoblotted for NRF2 and γTUBULIN proteins. B) Secreted levels of TNF-α cytokine were measured on the supernatants recovered at 24 hours by ELISA. Representative blots are shown from three independent experiments. Data reflect the mean ± SEM from the pool of three independent experiments. Significance was determined using Student’s t test. *** p < 0.001.
(TIF)

S6 Fig Related to Fig 6. **The NRF2 pathway is initiated by contact of parasites with its host cell in *Leishmania* infection**. A and B) BMDMs from WT, *Nox2*^-/-^ and *Nrf2*^-/-^ mice were either non-stimulated (Ø) or infected with live or PFA-fixed (A), or UV-treated (B) *Lgy*LRV1+ or *Lgy*LRV1 parasites, or stimulated with tBHQ (10 μM) for 4 hrs. Cells lysates were immunoblotted for NRF2 and γTUBULIN proteins. C) BMDMs from WT, *Nox2*^-/-^, *Inos*^-/-^ and *Nrf2*^-/-^ were infected with live or PFA-fixed *Lmj* or *Linf* S1 or *Linf* S2 parasites. Negative and positive controls for NRF2 activation of non-treated (Ø), or tBHQ-treated (10 μM) were performed concurrently. Cells were lysed at 4 hrs and were immunoblotted for NRF2 and γTUBULIN proteins. D) WT cells were either infected with live, or heat-killed, or UV-treated or PFA-fixed *Lgy*LRV1+ parasites for 8 hrs. Non-treated (Ø) or tBHQ-treated (10 μM) or β-glucan peptide (BGP)-treated (100 *μ*g/ml) cells were performed concurrently. Relative RNA expression levels of *Nqo1 gene* were normalized to *L32* housekeeping gene assessed by RT-qPCR using the 2^-ΔΔCT^ method. E) WT cells were either infected with live, or heat-killed, or UV-treated or PFA-fixed *Lgy*LRV1+ parasites for 24 hrs. Secreted levels of TNF-α cytokine were measured on the supernatants recovered by ELISA. F) WT and *Nrf2*^-/-^ cells were either non-stimulated (Ø), or infected with *Lgy*LRV1+ parasites, or zymosan particle-treated (1 *μ*g/ml) for 4 hrs. Cell lysates after 4 hrs post-infection were analyzed by Western Blot using anti-NRF2 and anti-γTUBULIN. G) WT cells were either infected with *Lgy*LRV1+ parasites or non-stimulated (Ø) for 15 min. DNA (blue) was stained with DAPI for assessing parasite internalization at 63x using confocal microscopy. Scale bar represents 10 μm. Representative blots and images from three (A-B), two (C and F) or one (G) independent experiments are shown. Data reflect the mean ± SEM from the pool of three independent experiments. Not significant (ns), * p < 0.05, ** p < 0.01 and *** p < 0.001.
(TIF)

S7 Fig Related to Fig 7. **SFKs are responsible for NRF2 signaling in *Lgy* infection**. A and C) WT, *Nox2*^-/-^ and *Nrf2*^-/-^ BMDMs were pretreated with DMSO (-), PP2 (100 μM) (A) or PP3 (10 μM) (C) for 1 hr and infected with either *Lgy*LRV1+ or *Lgy*LRV1- parasites, or treated with tBHQ (10 μM), or non-treated (Ø). Cell lysates after 8 hrs were analyzed by Western Blot using anti-phospho-NRF2 (S40), anti-NRF2, anti-phospho-PKCδ (Y311), anti-PKCδ, anti-phospho-SRC (Y416), anti-SRC and anti-γTUBULIN antibodies. B) WT cells were pretreated with DMSO (-) or PP2 (100 μM) for 1 hr and infected with either *Lgy*LRV1+ or *Lgy*LRV1- parasites, or non-infected (Ø) for 8 hrs. Intracellular parasite load was quantified by DAPI staining at 40x using a high-content microscope. Parasite load was quantified using MetaXpress software. D) WT cells were pretreated with DMSO (-) or KB SRC 4 (100 μM) for 1 hr and infected with either *Lgy*LRV1+ or *Lgy*LRV1- parasites, or non-infected (Ø) for 8 hrs. Relative RNA expression levels of the *Nrf2 gene* were normalized to *L32* housekeeping gene assessed by RT-qPCR using the 2^-ΔΔCT^ method. E) WT cells pretreated with DMSO (-) or KB SRC 4 (100 μM) for 1 hr and infected with *Lgy*LRV1+ parasites, or non-infected (Ø) for 24 hrs. Secreted levels of TNF-α cytokine were measured on the supernatants recovered by ELISA. F) WT and *Nrf2* ^-/-^ BMDMs were infected with *Lgy*LRV1+, *Lgy*LRV1-parasites for 1 to 8 hrs. Negative and positive controls for NRF2 activation of non-treated (Ø), or tBHQ-treated (10 μM). Cell lysates were analyzed by Western Blot using anti-phospho- eiF2α (S51), anti- eiF2α and anti-γTUBULIN antibodies. Representative blots from two (B and F) or three (A) independent experiments are shown. The graphs show pool data expressed as mean ± SEM from two (B and E) or three (D) independent experiments. Unpaired Student’s t test was used to calculate statistical significance. Not significant (ns), * p < 0.05, ** and *** p < 0.001.
(TIF)

S1 Table Oligonucleotides used in this study. Oligonucleotides used for either RT-qPCR, mice genotyping or mCherry parasites generation. Fw = Forward and Rev = Reverse.
(DOCX)

S2 Table Reagents used in this study. Reagents list indicating the source and the identifier.
(DOCX)

S3 Table Antibodies used in this study. Antibodies used for either Western Blot analysis, high-content microscopy or confocal microscopy. The source and the identifier are indicated and for some antibodies the Research Resource Identifier (RRID) is also added.
(DOCX)

S1 Data Data used in this study. Excel spreadsheet containing, in separate sheets, the underlying numerical data for Figs 1–7 and S1–S7.
(XLSX)
