# Supplementary figures and images for: The antioxidant response favors Leishmania parasites survival, limits inflammation and reprograms the host cell metabolism
Source: PLoS Pathog. 2021 Mar 25;17(3):e1009422. doi: 10.1371/journal.ppat.1009422 (PMC7993605; doi:10.1371/journal.ppat.1009422)

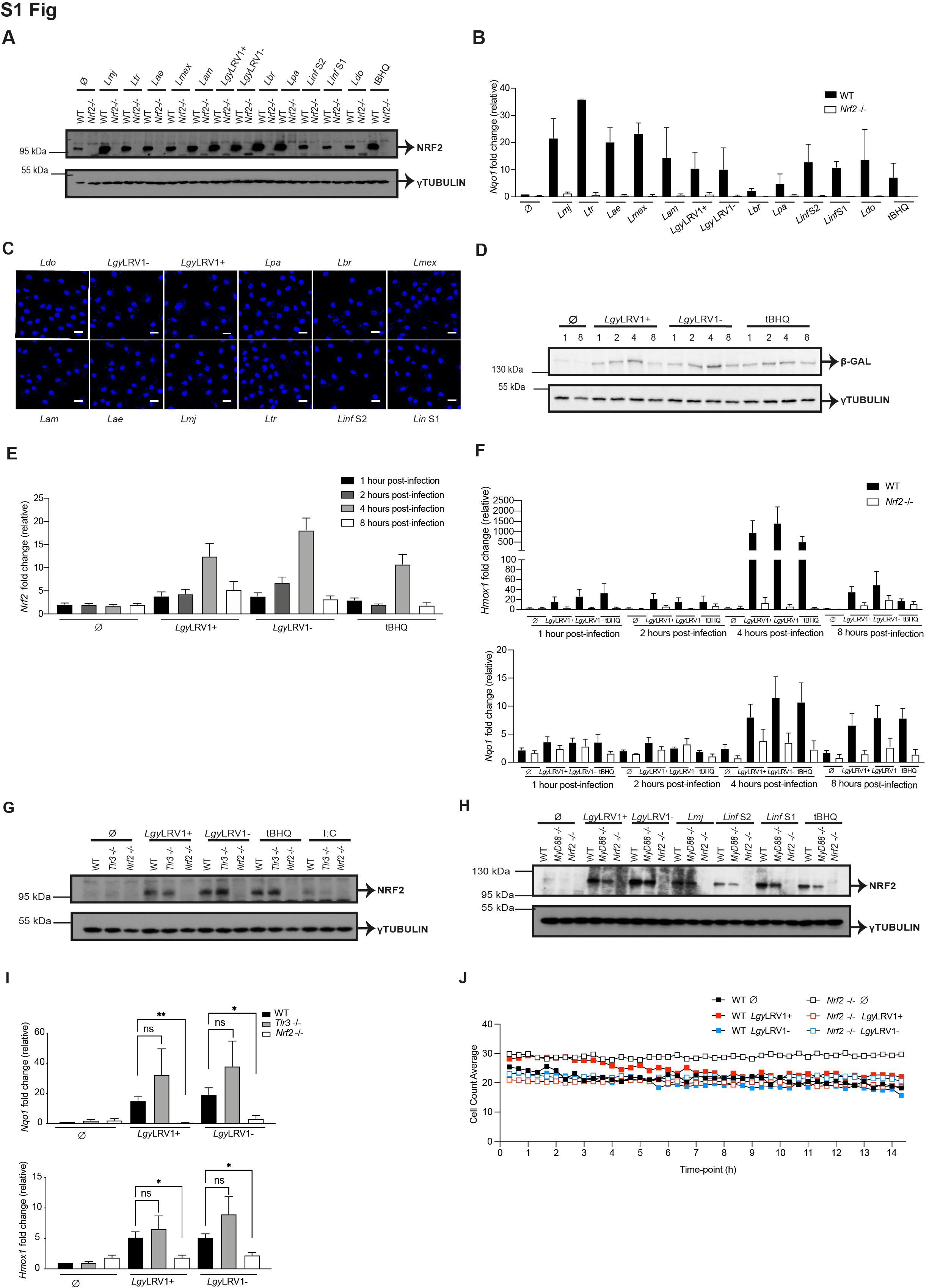

Supplement: S1 Fig — Lgy parasites are sufficient to upregulate the NRF2 pathway without LRV1 and L. spp. infection in NRF2 expression. A and B) WT and Nrf2 -/- BMDMs were infected with Lmj, Ltr, Lae, Lmex, Lam, LgyLRV1+, LgyLRV1-, Lbr, Lpa, Linf S1, Linf S2 or Ldo parasites. Negative and positive controls for NRF2 activation of non-treated (Ø), or tBHQ-treated (10 μM) were performed concurrently. NRF2 protein levels were assessed by Western Blot at 8 hrs using anti-NRF2 and anti-γTUBULIN (A) and Nqo1 relative RNA levels were normalized to L32 housekeeping gene assessed by RT-qPCR using the 2ΔΔCT method (B). C) WT cells were infected with Lmj, Ltr, Lae, Lmex, Lam, LgyLRV1+, LgyLRV1-, Lbr, Lpa, Linf S1, Linf S2 or Ldo parasites for 8 hrs. DNA (blue) was stained with DAPI for assessing parasite internalization at 63x using confocal microscopy. Scale bar represents 10 μm. D) Nrf2 -/- BMDMs were infected with LgyLRV1+, LgyLRV1-parasites. Negative and positive controls for NRF2 activation of non-treated (Ø), or tBHQ-treated (10 μM) were performed concurrently. β-galactosidase (β-GAL) protein levels were assessed by Western Blot using anti- β-GAL and anti- γTUBULIN antibodies at the indicated time-points. E) WT cells were infected with either LgyLRV1+ or LgyLRV1- or non-treated (Ø), or tBHQ-treated (10 μM) for 1, 2, 4 and 8 hr time-points. Nrf2 relative RNA levels were normalized to the L32 housekeeping gene assessed by RT-qPCR using the 2-ΔΔCT method. F) WT and Nrf2 -/- cells were infected with either LgyLRV1+ or LgyLRV1- or non-treated (Ø), or tBHQ-treated (10 μM) for 1, 2, 4 and 8 hr time-points. Hmox1 and Nqo1 relative RNA levels were normalized to L32 housekeeping gene assessed by RT-qPCR using the 2-ΔΔCT method. G and I) WT, Tlr3 -/- and Nrf2 -/- BMDMs were infected with either LgyLRV1+ or LgyLRV1- parasites. Negative and positive controls of non-treated (Ø), tBHQ (10 μM), poly I:C (I:C, 2 μg/ml), TLR3 agonist, were performed concomitantly. NRF2 protein levels were assessed by Western B [file ppat.1009422.s001.tif]

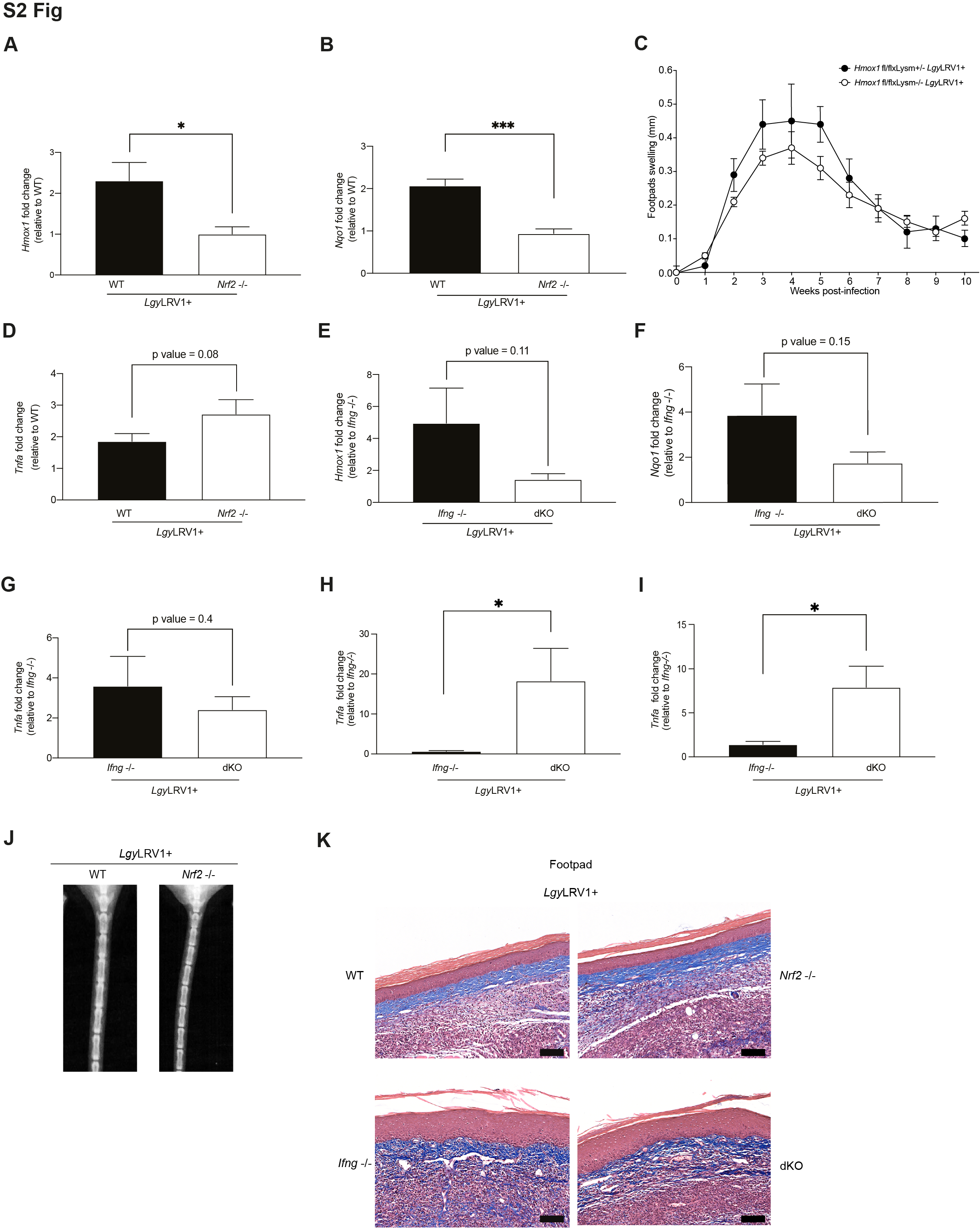

Supplement: S2 Fig — NRF2 controls inflammation in LgyLRV1+ infection by blocking proinflammatory cytokine Tnfa transcripts. WT, Nrf2-/-, Ifng-/-, IfngxNrf2 dKO (dKO), Hmox1fl/flxLysm+/- or Hmox1fl/flxLysm-/- mice were infected in both hind footpads with 1x10^6^ of LgyLRV1+ parasites. A and B) Relative RNA expression levels of NRF2 target genes Hmox1 (A) and Nqo1 (B) in the footpads of WT and Nrf2-/- at 3 weeks post-infection using RT-qPCR normalized to L32 housekeeping gene. C) Footpad swelling evolution was measured weekly as a proxy for disease progression for Hmox1fl/flxLysm+/- or Hmox1fl/flxLysm-/-. D) Relative RNA expression levels of Tnfa transcripts in the footpads of WT and Nrf2-/- at 3 weeks post-infection using RT-qPCR normalized to L32 housekeeping gene. E-G) Relative RNA expression levels of Hmox1 (E), Nqo1 (F) and Tnfa transcripts (G) in the footpads of Ifng-/- and dKO at 3 weeks post-infection using RT-qPCR normalized to L32 housekeeping gene. H and I) Relative RNA expression levels of Tnfa transcripts in the footpads (H) or tail (I) of Ifng-/- and dKO at 8 weeks post-infection using RT-qPCR normalized to L32 housekeeping gene. J) Representative X-Ray images indicating tail tissue and bone destruction at week 8 for WT and Nrf2-/- mice. K) Representative images of Masson’s trichrome staining of footpad sections at week 3 for WT, Nrf2-/-, Ifng-/- and dKO mice at 20x on an automated slide scanning microscope. Collagen (blue), nuclei (black), muscle and cytoplasm (red) are represented. Scale bar represents 100 μm. Data show mean ± SEM from the pool of one (A-G and K) two (H and I) or three (J) independent experiments (n = 5–12 mice). Significance was calculated by Student’s t test. * p < 0.05 and *** p < 0.001. (TIF) [file ppat.1009422.s002.tif]

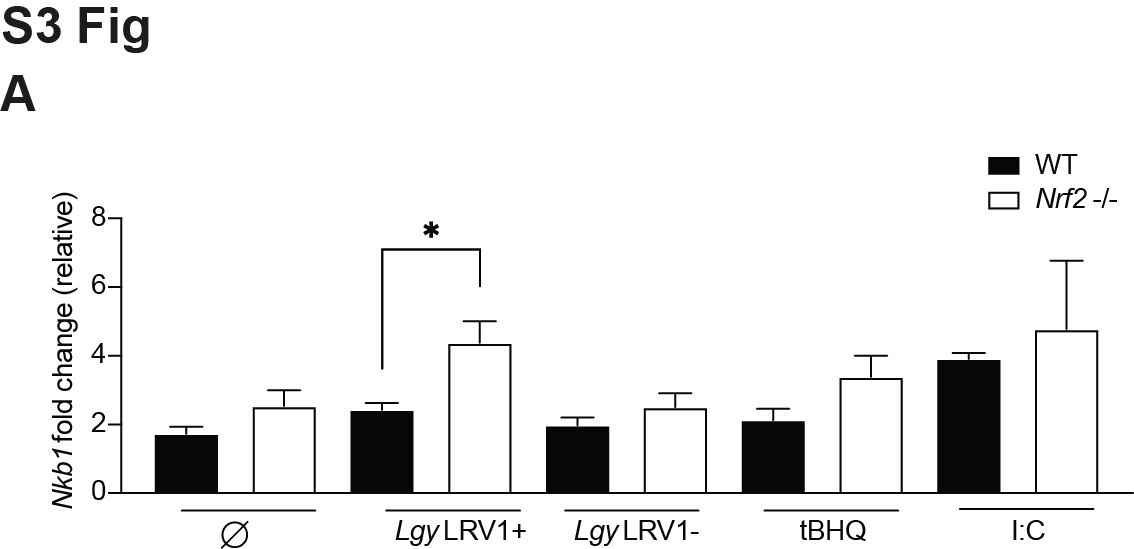

Supplement: S3 Fig — NRF2 expression controls NF-κB -inflammation in LgyLRV1+ infected macrophages. Relative RNA expression levels of Nfkb1 gene at 8 hrs in WT and Nrf2 -/- cells infected with either LgyLRV1+ or LgyLRV1- parasites, non-treated (Ø), tBHQ-treated (10 μM), or poly I:C (I:C, 2 μg/ml), assessed by RT-qPCR and normalized to the L32 housekeeping gene. Data expressed as mean ± SEM from two independent experiments. Unpaired Student’s t test was used to measure statistical significance. * p < 0.05. (TIF) [file ppat.1009422.s003.tif]

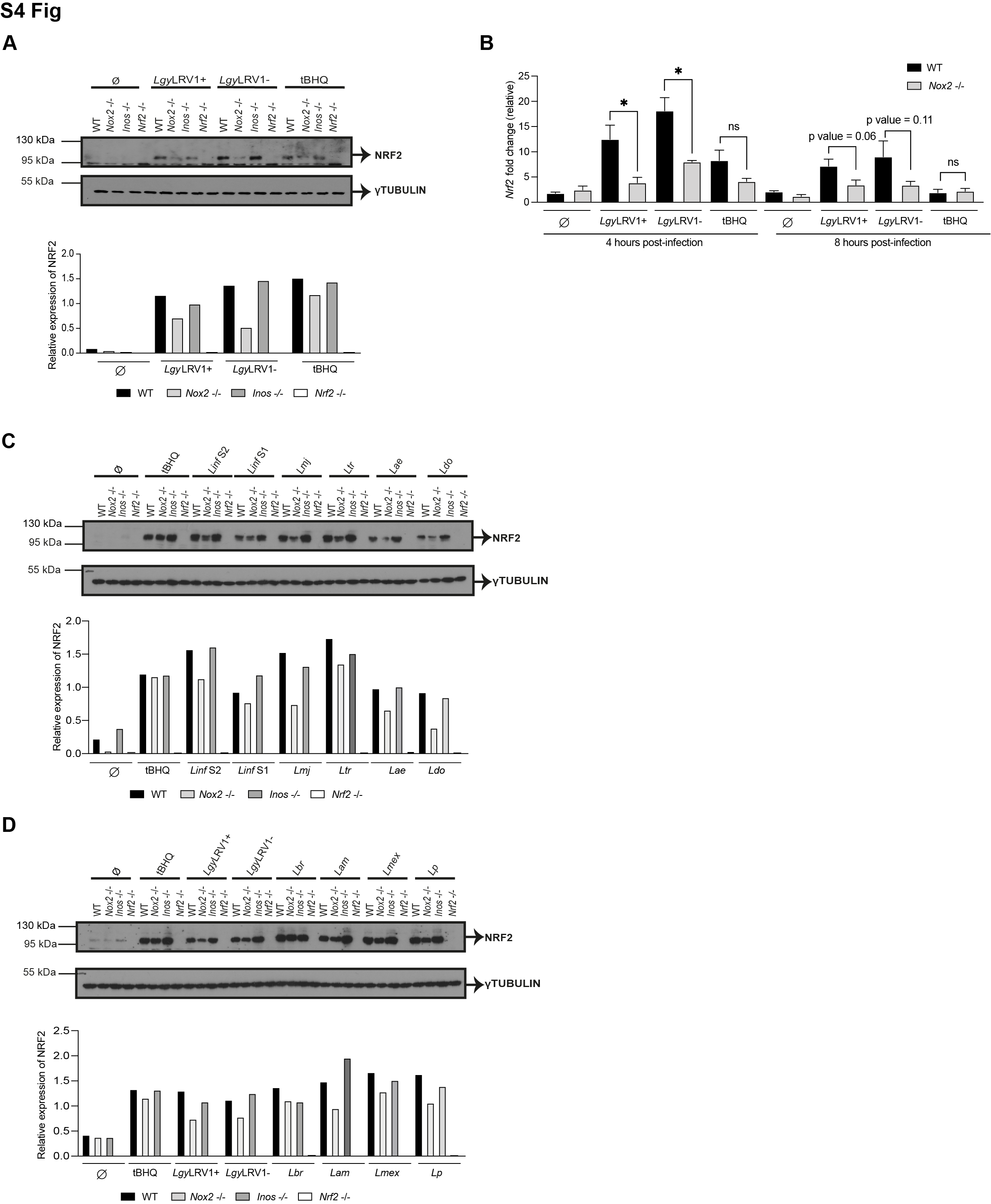

Supplement: S4 Fig — NOX2 regulation of the NRF2 pathway is conserved along Leishmania parasites. BMDMs from WT, Nox2 -/-, Inos -/- and Nrf2 -/- mice were infected with either Lmj, Ltr, Lae, Lmex, Lam, LgyLRV1+, LgyLRV1-, Lbr, Lpa, Linf S1, Linf S2 or Ldo parasites. Negative and positive controls for NRF2 activation of non-treated (Ø), or tBHQ-treated (10 μM) were performed concurrently. A, C and D) Immunoblotting of NRF2 and γTUBULIN proteins in cell lysates at 4 (B and C) and 8 (A) hrs. Relative NRF2 levels were determined by band quantification using Image J software and given as NRF2 over γTUBULIN. B) Relative RNA expression levels of Nrf2 were normalized to L32 housekeeping gene assessed by RT-qPCR using the 2-ΔΔCT method in WT and Nox2 -/- cells. Representative blots and their quantification from three independent experiments are shown. Data expressed as mean ± SEM from two independent experiments. Unpaired Student’s t test was used to measure statistical significance. Not significant (ns) and * p < 0.05. (TIF) [file ppat.1009422.s004.tif]

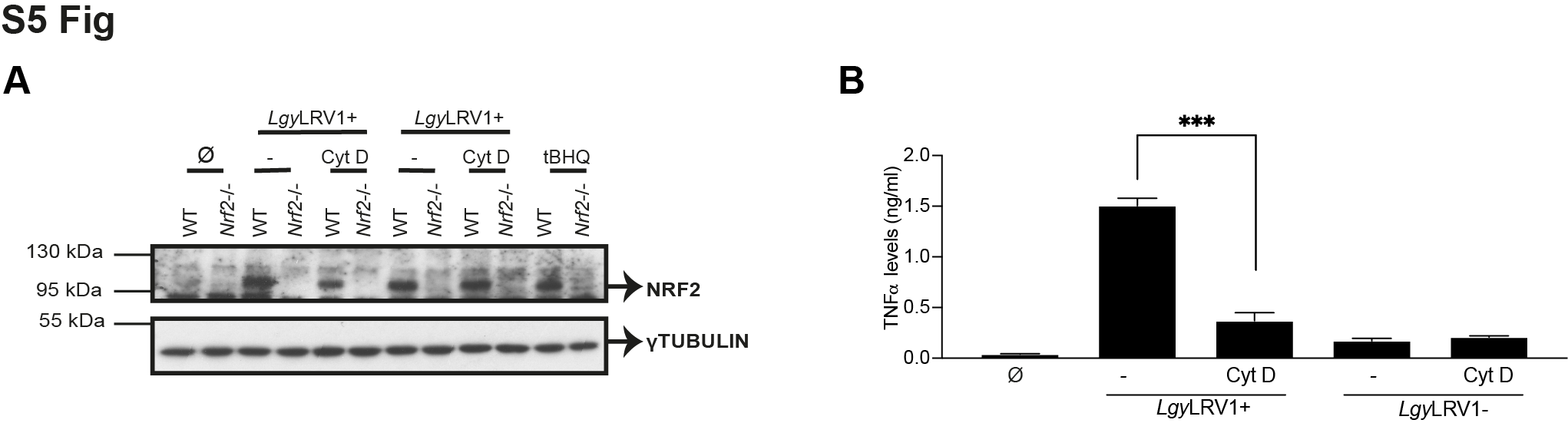

Supplement: S5 Fig — The NRF2 pathway is not abrogated by phagocytosis blockage in Lgy infection. A and B) WT cells were pretreated with DMSO or Cytochalasin D (Cyt D, 40 μM) for 1 hr and infected with LgyLRV1+ or LgyLRV1- parasites or stimulated with tBHQ (10 μM) or medium-treated (Ø). A) Cells were lysed at the 8 hr time-point and immunoblotted for NRF2 and γTUBULIN proteins. B) Secreted levels of TNF-α cytokine were measured on the supernatants recovered at 24 hours by ELISA. Representative blots are shown from three independent experiments. Data reflect the mean ± SEM from the pool of three independent experiments. Significance was determined using Student’s t test. *** p < 0.001. (TIF) [file ppat.1009422.s005.tif]

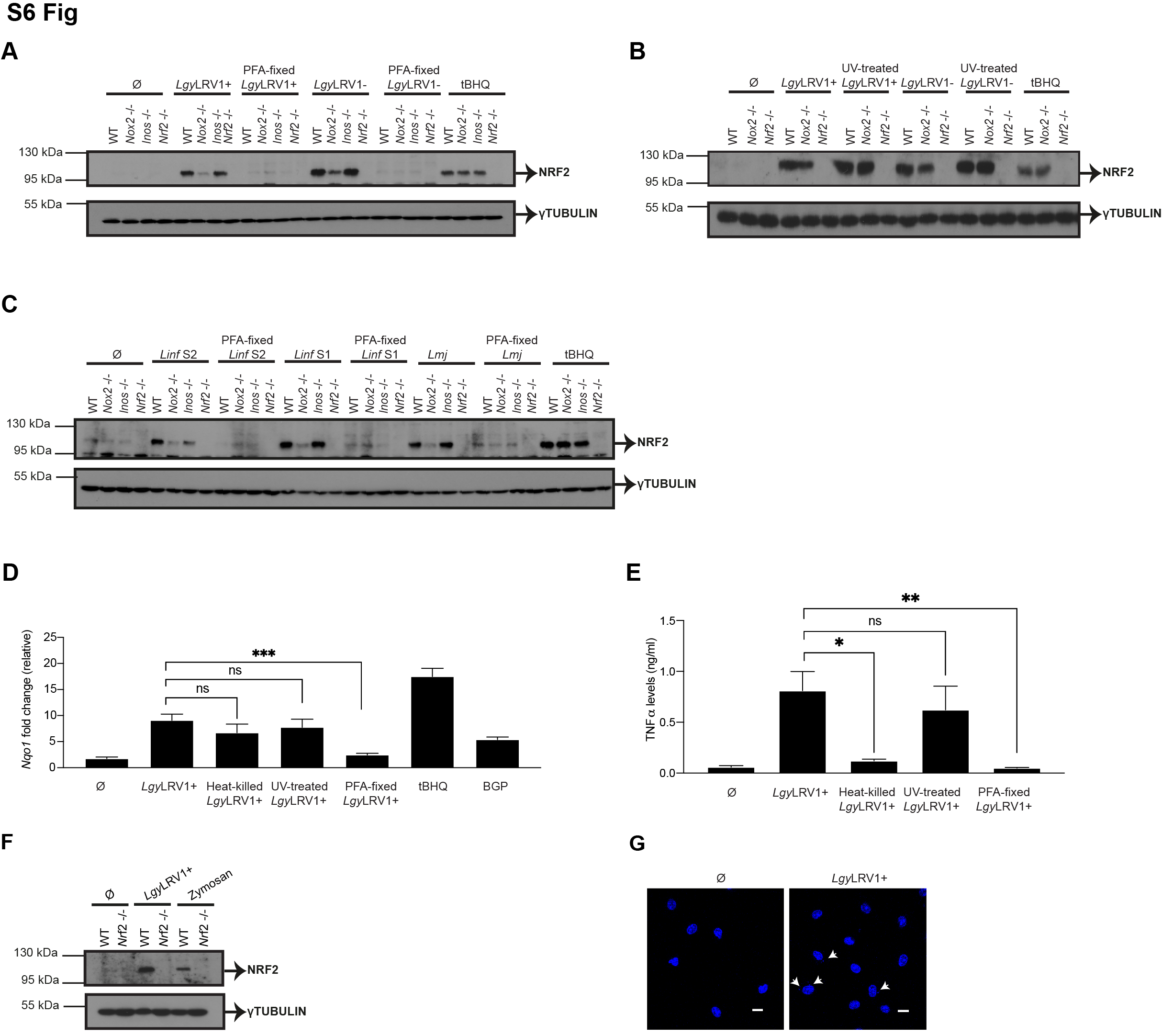

Supplement: S6 Fig — The NRF2 pathway is initiated by contact of parasites with its host cell in Leishmania infection. A and B) BMDMs from WT, Nox2-/- and Nrf2-/- mice were either non-stimulated (Ø) or infected with live or PFA-fixed (A), or UV-treated (B) LgyLRV1+ or LgyLRV1 parasites, or stimulated with tBHQ (10 μM) for 4 hrs. Cells lysates were immunoblotted for NRF2 and γTUBULIN proteins. C) BMDMs from WT, Nox2-/-, Inos-/- and Nrf2-/- were infected with live or PFA-fixed Lmj or Linf S1 or Linf S2 parasites. Negative and positive controls for NRF2 activation of non-treated (Ø), or tBHQ-treated (10 μM) were performed concurrently. Cells were lysed at 4 hrs and were immunoblotted for NRF2 and γTUBULIN proteins. D) WT cells were either infected with live, or heat-killed, or UV-treated or PFA-fixed LgyLRV1+ parasites for 8 hrs. Non-treated (Ø) or tBHQ-treated (10 μM) or β-glucan peptide (BGP)-treated (100 μg/ml) cells were performed concurrently. Relative RNA expression levels of Nqo1 gene were normalized to L32 housekeeping gene assessed by RT-qPCR using the 2-ΔΔCT method. E) WT cells were either infected with live, or heat-killed, or UV-treated or PFA-fixed LgyLRV1+ parasites for 24 hrs. Secreted levels of TNF-α cytokine were measured on the supernatants recovered by ELISA. F) WT and Nrf2-/- cells were either non-stimulated (Ø), or infected with LgyLRV1+ parasites, or zymosan particle-treated (1 μg/ml) for 4 hrs. Cell lysates after 4 hrs post-infection were analyzed by Western Blot using anti-NRF2 and anti-γTUBULIN. G) WT cells were either infected with LgyLRV1+ parasites or non-stimulated (Ø) for 15 min. DNA (blue) was stained with DAPI for assessing parasite internalization at 63x using confocal microscopy. Scale bar represents 10 μm. Representative blots and images from three (A-B), two (C and F) or one (G) independent experiments are shown. Data reflect the mean ± SEM from the pool of three independent experiments. Not significant (ns), * p < 0.05, ** p < 0.01 and *** p < 0.001. (T [file ppat.1009422.s006.tif]

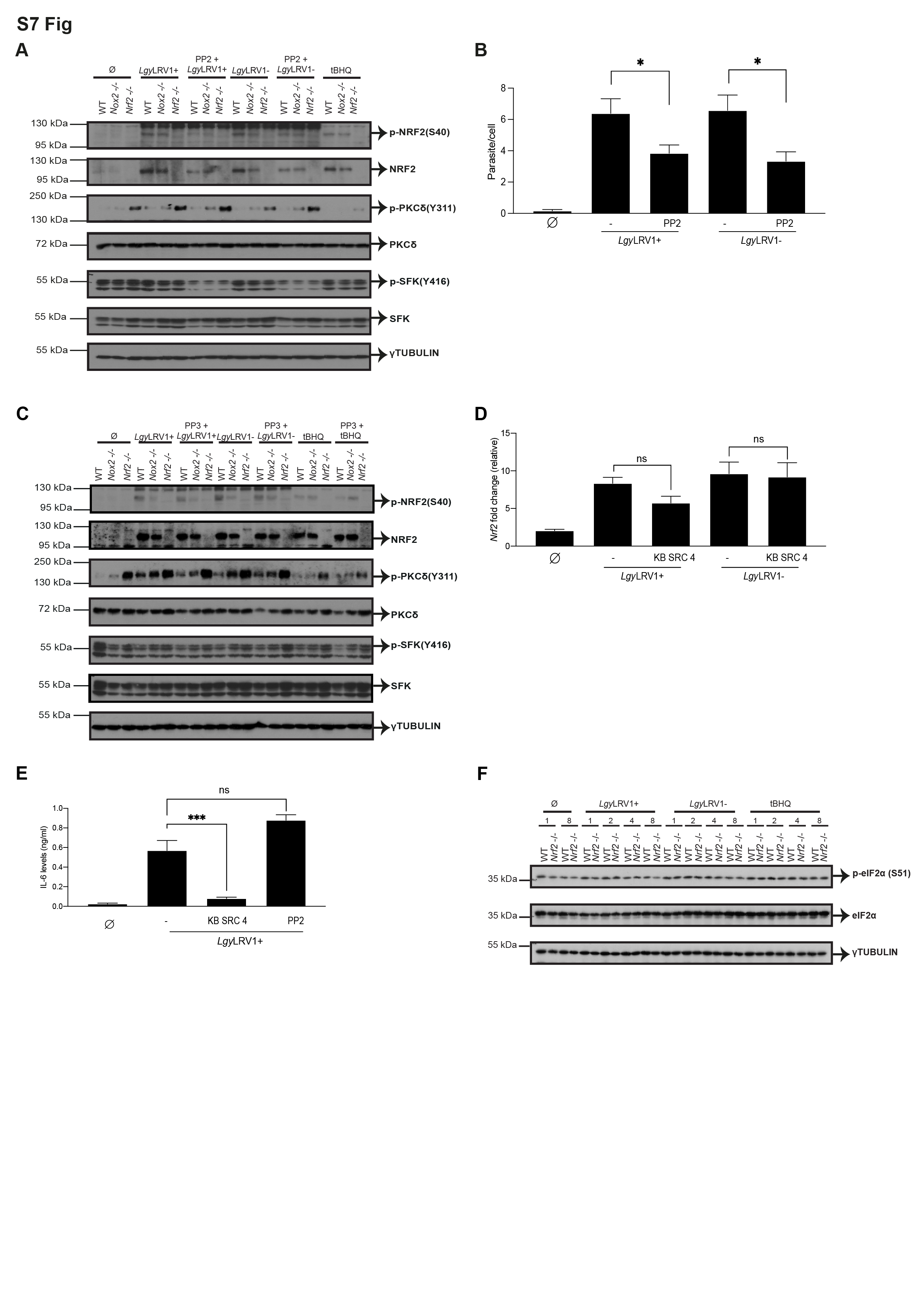

Supplement: S7 Fig — SFKs are responsible for NRF2 signaling in Lgy infection. A and C) WT, Nox2-/- and Nrf2-/- BMDMs were pretreated with DMSO (-), PP2 (100 μM) (A) or PP3 (10 μM) (C) for 1 hr and infected with either LgyLRV1+ or LgyLRV1- parasites, or treated with tBHQ (10 μM), or non-treated (Ø). Cell lysates after 8 hrs were analyzed by Western Blot using anti-phospho-NRF2 (S40), anti-NRF2, anti-phospho-PKCδ (Y311), anti-PKCδ, anti-phospho-SRC (Y416), anti-SRC and anti-γTUBULIN antibodies. B) WT cells were pretreated with DMSO (-) or PP2 (100 μM) for 1 hr and infected with either LgyLRV1+ or LgyLRV1- parasites, or non-infected (Ø) for 8 hrs. Intracellular parasite load was quantified by DAPI staining at 40x using a high-content microscope. Parasite load was quantified using MetaXpress software. D) WT cells were pretreated with DMSO (-) or KB SRC 4 (100 μM) for 1 hr and infected with either LgyLRV1+ or LgyLRV1- parasites, or non-infected (Ø) for 8 hrs. Relative RNA expression levels of the Nrf2 gene were normalized to L32 housekeeping gene assessed by RT-qPCR using the 2-ΔΔCT method. E) WT cells pretreated with DMSO (-) or KB SRC 4 (100 μM) for 1 hr and infected with LgyLRV1+ parasites, or non-infected (Ø) for 24 hrs. Secreted levels of TNF-α cytokine were measured on the supernatants recovered by ELISA. F) WT and Nrf2 -/- BMDMs were infected with LgyLRV1+, LgyLRV1-parasites for 1 to 8 hrs. Negative and positive controls for NRF2 activation of non-treated (Ø), or tBHQ-treated (10 μM). Cell lysates were analyzed by Western Blot using anti-phospho- eiF2α (S51), anti- eiF2α and anti-γTUBULIN antibodies. Representative blots from two (B and F) or three (A) independent experiments are shown. The graphs show pool data expressed as mean ± SEM from two (B and E) or three (D) independent experiments. Unpaired Student’s t test was used to calculate statistical significance. Not significant (ns), * p < 0.05, ** and *** p < 0.001. (TIF) [file ppat.1009422.s007.tif]
