# Supplementary material for: The antioxidant response favors Leishmania parasites survival, limits inflammation and reprograms the host cell metabolism
Source: PLoS Pathog. 2021 Mar 25;17(3):e1009422. doi: 10.1371/journal.ppat.1009422 (PMC7993605; doi:10.1371/journal.ppat.1009422)
Supplement: S1 Table — Oligonucleotides used for either RT-qPCR, mice genotyping or mCherry parasites generation. Fw = Forward and Rev = Reverse. (DOCX) [file ppat.1009422.s008.docx]

**S1 Table. Oligonucleotides used in this study**

| **Primer name** | **Sequence 5’-3’** |
| --- | --- |
| **Primers for RT-qPCR** | |
| *Hmox1* Fw | ACATCGACAGCCCCACCAAGTTCAA |
| *Hmox1* Rev | CTGACGAAGTGACGCCATCTGTGAG |
| *Nqo1* Fw | AGGATGGGAGGTACTCGAATC |
| *Nqo1* Rev | TGCTAGAGATGACTCGGAAGG |
| *L32* Fw | AAGCGAAACTGGCGGAAAC |
| *L32* Rev | TAACCGATGTTGGGCATCAG |
| *Tnfa* Fw | CATCTTCTCAAA ATTCGAGTGACAA |
| *Tnfa* Rev | TGGGAGTAGACAAGGTACAACCC |
| *Nfkb1 Fw* | CCGCCTTCTGCTTGTAGATAG |
| *Nfkb1 Rev* | ATTTGATAACACTGGAAGCACGG |
| **Primers for mice genotyping** | |
| *Ifng* Mutant Fw | CCTTCTATCGCC TTCTTGACG |
| *Ifng* WT Fw | AGAAGTAAGTGGAAGGGCCCAGAAG |
| *Ifng* Common | AGGGAAACTGGGAGAGGAGAAATAT |
| *Nrf2* Mutant Rev | GCGGATTGACCGTAATGGGATAGG |
| *Nrf2* WT Rev | GCCGCCTTTTCAGTAGATGGAGG |
| *Nrf2* Common | TGGACGGGACTATTGAAGGCTG |
| **Primers for mCherry ORF** | |
| SMB7961 | GATCAGATCTCCACCATGGTGAGCAAGGGCGAGGAGG |
| SMB7962 | GATCAGATCTCTACTTGTACAGCTCGTCCAT |
