# Supplementary material for: The antioxidant response favors Leishmania parasites survival, limits inflammation and reprograms the host cell metabolism
Source: PLoS Pathog. 2021 Mar 25;17(3):e1009422. doi: 10.1371/journal.ppat.1009422 (PMC7993605; doi:10.1371/journal.ppat.1009422)
Supplement: S2 Table — Reagents list indicating the source and the identifier. (DOCX) [file ppat.1009422.s009.docx]

**S2 Table. Reagents used in this study**

| **Reagent** | **Source** | **Identifier** |
| --- | --- | --- |
| **Chemicals** | | |
| Recombinant Mouse Macrophage Colony Stimulating Factor (rm M-CSF) | ImmunoTools | Cat# 12343117 |
| Luminol sodium salt | Carbosynth | Cat# FL02733 |
| Donkey Serum | BIO-RAD | Cat# C06SB |
| VivoGlo Luciferin, In Vivo Grade | Promega | Cat# P1043 |
| Bovine Serum Albumin (BSA) | Sigma-Aldrich | Cat# A2153 |
| Hydrogen peroxide (H_2_O_2_) solution 30% (w/w) in H_2_O | Sigma-Aldrich | Cat# H1009 |
| PP3 | Tocris | Cat# 2794 |
| *tert*-Butylhydroquinone (tBHQ) | Sigma-Aldrich | Cat# **112941** |
| Cytochalasin D | Sigma-Aldrich | Cat# C8273 |
| TRI Reagent | Molecular Research Center, inc | Cat# TR 118 |
| PRImeZOL Reagent | Canvax | Cat# AN1100 |
| SuperScript II Reverse Transcriptase | Invitrogen | Cat#18064022 |
| LightCycler 480 SYBR Green I Master | Roche | Cat# 04707516001 |
| DAPI (4',6-Diamidino-2-Phenylindole, Dihydrochloride) | Invitrogen | Cat# D1306 |
| PP2 | Sigma-Aldrich | Cat# P0042 |
| MK2206 | Apexbio | Cat# A3010 |
| KB SRC 4 | R&D Systems | Cat# 4660 |
| Dimethyl sulfoxide (DMSO) Cell culture grade | AppliChem GmbH | Cat# A3672 |
| Seahorse XF DMEM Medium, pH 7.4 | Bucher Biotek | Cat# 103575-100 |
| Seahorse XFp Fluk Pak | Bucher Biotek | Cat# 103025-100 |
| Schneider’s Drosophila Medium | PAN BIOTECH | Cat# P04-91500 |
| Fetal Bovine Serum (FBS) | Gibco | Cat# 10270106 |
| Hemin BioXtra, from Porcine | Sigma-Aldrich | Cat# 51280 |
| Folic acid | Fluka | Cat# 47620 |
| HEPES Buffer (1M) | BioConcept | Cat# 5-31F00-H |
| 6-Biopterin | Sigma-Aldrich | Cat# B2517 |
| Penicillin-Streptomycin (P/S) solution | BioConcept | Cat# 4-01F00-H |
| Dulbecco's Phosphate-Buffered Saline (DPBS) | Gibco | Cat# 14040091 |
| Dulbecco’s modified Eagle’s medium (DMEM) | Gibco | Cat# [31966021](http://www.bio-thing.com/p115324) |
| DMEM, high glucose, HEPES, no phenol red | Gibco | Cat# 21063029 |
| RPMI 1640 Medium | Gibco | Cat# 61870044 |
| Medium 199 (M199) | Gibco | Cat# 11150 |
| Saponin | Sigma-Aldrich | Cat# 84510 |
| Dihydrorhodamine 123 | Invitrogen | Cat# D23806 |
| Poly (I :C) HMW | InvivoGen | Cat# tlrl-pic |
| Shandon TBD-1 Decalcifier | Thermofisher Scientific | Cat# 6764001 |
| cOmplete, Mini, EDTA-free Protease Inhibitor Cocktail | Roche | Cat# **11836170001** |
| 5x RIPA Buffer IV with Triton-X-100 (pH 7.4) | Bio Basic | Cat# RB4478 |
| Triton X-100 | Roche | Cat# 10789704001 |
| Paraformaldehyde | Fluka | Cat# P6148 |
| Zymosan | Invivogen | Cat# tlrl-zym |
| Beta-glucan peptide (BGP) | Invivogen | Cat# tlrl-bgp |
| **Critical Commercial Assays** | | |
| CFSE Fluorescent Cell Labeling Kit | Abcam | Cat# ab113853 |
| TNF-$\alpha$ Mouse Uncoated ELISA Kit | Invitrogen | Cat# 88-7324-88 |
| IL-6 Mouse Uncoated ELISA Kit | Invitrogen | Cat# 88-7064-88 |
| KAPA Mouse Genotyping Kit | KAPA BIOSYSTEMS | Cat# KK7301 |
| Direct-zol-96 RNA | Zymo Research | Cat# R2054 |
| Pierce BCA Protein Assay Kit | Thermofisher Scientific | Cat# 23227 |
