# Supplementary material for: The antioxidant response favors Leishmania parasites survival, limits inflammation and reprograms the host cell metabolism
Source: PLoS Pathog. 2021 Mar 25;17(3):e1009422. doi: 10.1371/journal.ppat.1009422 (PMC7993605; doi:10.1371/journal.ppat.1009422)
Supplement: S3 Table — Antibodies used for either Western Blot analysis, high-content microscopy or confocal microscopy. The source and the identifier are indicated and for some antibodies the Research Resource Identifier (RRID) is also added. (DOCX) [file ppat.1009422.s010.docx]

**S3 Table. Antibodies used in this study**

| **Antibodies** | **Source** | **Identifier** |
| --- | --- | --- |
| Goat anti-mouse IgG (H+L) HRP Conjugate Antibody | Promega | Cat# W4021;  RRID: AB_430834 |
| Goat anti-rabbit IgG (H+L) HRP Conjugate Antibody | Promega | Cat# W4011;  RRID: AB_430833 |
| Mouse Anti-β-Galactosidase Monoclonal Antibody | Promega | Cat# Z3781;  RRID: AB_430877 |
| Rabbit Anti-NRF2 Monoclonal Antibody | Abcam | Cat# ab62352;  RRID: AB_944418 |
| Mouse Anti-γ-TUBULIN Monoclonal Antibody | Sigma-Aldrich | Cat# T5326;  RRID: AB_532292 |
| Mouse Anti-LAMIN A/C Monoclonal Antibody | Abcam | Cat# ab8984;  RRID: AB_306913 |
| Donkey Anti-Rabbit IgG (H+L) Alexa Fluor 555 Antibody | Invitrogen | Cat# A-31572;  RRID: AB_162543 |
| Donkey Anti-Mouse IgG (H+L) Alexa Fluor 488 Antibody | Invitrogen | Cat# A-21202;  RRID: AB_141607 |
| Rabbit Anti-Phospho NRF2 (S40) Polyclonal Antibody | BIOMATIK | Cat# CAF17554 |
| Rabbit Anti-SRC Polyclonal Antibody | Cell Signaling | Cat# 2108;  RRID: AB_331137 |
| Rabbit Anti-Phospho SRC Family (T416) Monoclonal Antibody | Cell Signaling | Cat# 6943;  RRID: AB_10013641 |
| Rabbit Anti-PKCδ Polyclonal Antibody | Cell Signaling | Cat# 2058;  RRID: AB_10694655 |
| Rabbit Anti-Phospho PKCδ (T311) Polyclonal Antibody | Cell Signaling | Cat# 2055 |
| Rabbit Anti-NF$\kappa$B P65 Monoclonal Antibody | Cell Signaling | Cat# 8242; RRID:  AB_10859369 |
| Rabbit Anti-AKT Monoclonal Antibody | Cell Signaling | Cat# 4691; RRID: AB_915783 |
| Rabbit Anti-Phospho AKT (T308) Monoclonal Antibody | Cell Signaling | Cat# 2965; RRID: AB_2255933 |
| Rabbit Anti-eiF2$\alpha$ Monoclonal Antibody | Cell Signaling | Cat# 5324 |
| Rabbit Anti-Phospho eiF2$\alpha$(S51) Monoclonal Antibody | Cell Signaling | Cat# 3597 |
